# Thioester-Containing Benzoate Derivatives with α-Glucosidase Inhibitory Activity from the Deep-Sea-Derived Fungus *Talaromyces indigoticus* FS688

**DOI:** 10.3390/md20010033

**Published:** 2021-12-29

**Authors:** Mingqiong Li, Saini Li, Jinhua Hu, Xiaoxia Gao, Yanlin Wang, Zhaoming Liu, Weimin Zhang

**Affiliations:** 1 State Key Laboratory of Applied Microbiology Southern China, Guangdong Provincial Key Laboratory of Microbial Culture Collection and Application, Institute of Microbiology, Guangdong Academy of Sciences, 100 Central Xianlie Road, Yuexiu District, Guangzhou 510070, China; LM_qiong@163.com (M.L.); lisn@gdim.cn (S.L.); m15622119835@163.com (J.H.); 2 College of Pharmacy, Guangdong Pharmaceutical University, Guangzhou 510006, China; gaoxxia91@163.com; 3 Key Laboratory of Ocean and Marginal Sea Geology, South China Sea Institute of Oceanology, Innovation Academy of South China Sea Ecology and Environmental Engineering, Chinese Academy of Sciences, Guangzhou 510301, China; yanlinw@scsio.ac.cn

**Keywords:** thioester-containing benzoate, deep-sea-derived fungus, α-glucosidase inhibitory activity, docking study

## Abstract

Eurothiocins C–H (**1**–**6**), six unusual thioester-containing benzoate derivatives, were isolated from the deep-sea-derived fungus *Talaromyces indigoticus* FS688 together with a known analogue eurothiocin A (**7**). Their structures were elucidated through spectroscopic analysis and the absolute configurations were determined by X-ray diffraction and ECD calculations. In addition, compound **1** exhibited significant inhibitory activity against α-glucosidase with an IC_50_ value of 5.4 μM, while compounds **4** and **5** showed moderate effects with IC_50_ values of 33.6 and 72.1 μM, respectively. A preliminary structure–activity relationship is discussed and a docking analysis was performed.

## 1. Introduction

Organosulfur compounds, referring to sulfur-containing low-molecular-weight compounds including thiols, thioesters, and sulfoxides, continue to be a research hotspot in the field of organic chemistry due to their unique chemical properties and reactive functions [1,2,3,4]. On the one hand, sulfur is an essential element in primary metabolism from bacteria and fungi to plants and animals; for example, the cysteine is the most important amino acid in protein structures and in protein-folding pathways [5]. On the other hand, sulfur-containing secondary metabolites produced by plant, animals or microorganisms always exhibit significant biological activities such as anti-inflammatory [6], anticancer [7] and plant defense [8]. Several clinical drugs developed from natural products (NPs) are organosulfur compounds such as penicillin, cephalosporine and trabectedin (ET-743). However, sulfur-containing NPs are still relatively rare when compared to other types of NPs, and the biological significance and biosynthetic mechanisms of sulfur-containing NPs have been investigated with limited progress [7].

Since sulfur is the second most common non-metallic element, after chlorine in sea water, sulfur-containing metabolites are widely produced by different marine organisms [9]. Marine microorganisms are potential and reproducible sources of new bioactive sulfur-containing NPs, and have attracted research interest worldwide in recent years. Since the first sulfur-containing metabolite, gliovictin, was discovered from marine deuteromycete *Asteromyces cruciatus* [10], 484 non-sulfated organosulfur NPs have been isolated from marine microorganisms as of the end of 2020 and fungi contributed the most significant number of the new compounds (43%), which were reviewed by Shao C.-L. et al. [11]. Thus, it could be concluded that marine fungi have become the most productive marine microorganisms of sulfur-containing NPs.

**Figure 1:**
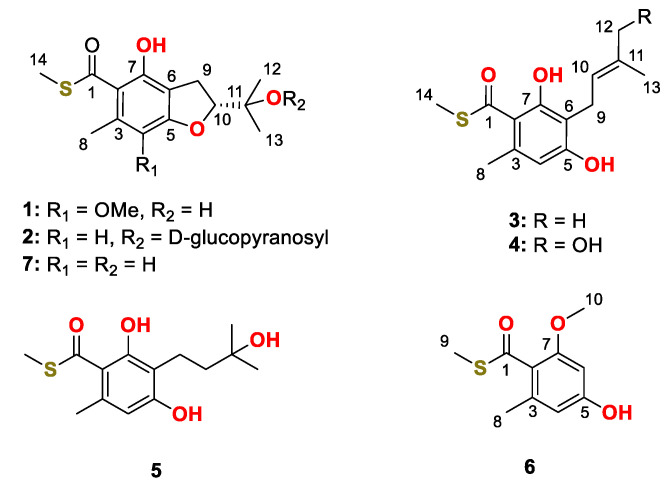
Chemical structures of compound **1**–**7**.

Deep-sea-derived fungi are a special group of microorganisms collected from sediment or water at a depth of over 1000 m. Due to their potential ability to produce novel and bioactive natural products promoted by the extreme environment, deep-sea-derived fungi have attracted considerable attention from both natural product and medicinal chemists [12]. In the course of searching for bioactive metabolites from deep-sea-derived fungi, *Talaromyces indigoticus* FS688—isolated from the South China Sea—was investigated and seven unusual thioester-containing benzoate derivatives were isolated (Figure 1). Herein, the details of the isolation, the structure identification and the biological evaluation of compounds **1**–**7** are discussed.

## 2. Results

### 2.1. Structure Identification

The fermented substrate of the fungus *Talaromyces indigoticus* FS688 was extracted with ethyl acetate three times and then concentrated under reduced pressure to give a brown oil, which was further subjected to repeated silica gel with gradient elution followed by Sephdex-20 and semi-preparative HPLC purification to afford compounds **1**−**7** (Figure 1).

Eurothiocin C (**1**) was obtained as a colorless oil. The molecular formula was deduced to be C_15_H_19_O_5_S on the basis of the deprotonated quasi molecular ion peak at *m/z* 311.0964 [M – H]^−^ and the characteristic ^34^S-containing isotope ion peak at *m/z* 313.0938 [M – H + 2]^−^ in a 20:1 ratio with the ^32^S-containing ion peak from the HRESIMS spectrum. The ^1^H NMR spectrum (Table 1) showed the signals of a chelating proton at *δ*_H_ 11.14 (7-OH), five singlet methyls including a methoxy group at *δ*_H_ 3.78 (Me-18), and intercoupled methylene and methine groups at *δ*_H_ 3.17/3.09 (H_2_-9) and *δ*_H_ 4.78 (H-10), respectively. The ^13^C NMR data resolved 15 resonances composed of six aromatic carbons, eight aliphatic carbons and a carbonyl carbon (*δ*_C_ 197.8), suggesting that a fully substituted benzene ring should be contained in compound **1**. By comparing the 1D NMR data to that of the known compound eurothiocin A (**7**), [13] indicated that compound **1** was a methoxy additive product of eurothiocin A.

**Figure 2:**
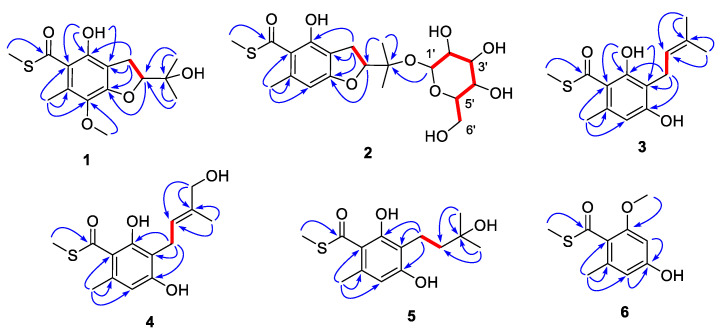
COSY (red bold lines) and key HMBC correlations (blue arrows) of compounds **1**–**6**.

The complete structure was elucidated by analysis of 2D NMR data (Figure 2). The COSY correlations of H_2_-9/H-10 and the HMBC correlations from H_3_-12/H_3_-13 to C-10/C-11 indicated the presence of an oxygenated isopentenyl unit (C-9 to C-13), which was further connected to C-6 of the benzene ring based on the correlations from H_2_-9 to C-5, C-6 and C-7. A benzofuran core was constructed by the key HMBC correlations from H-10 to C-5. The chelating proton at 7-OH displayed HMBC correlations to C-2, C-6 and C-7, which suggested that the carbonyl group should be linked to C-2 of the benzene ring to form an intramolecular hydrogen bond. Finally, the methyl thioester moiety was deduced by the correlations from H_3_-14 to C-1 and the characteristic chemical shifts of C-1 (*δ*_C_ 197.8), while the locations of Me-8 and 4-OMe were evaluated by the cross-peaks from H_3_-8 to C-2/C-3/C-4 and from 4-OMe to C-4. Hence, the planar structure of compound **1** was elucidated to be a sulfur-containing benzofuran derivative as shown.

**Figure 3:**
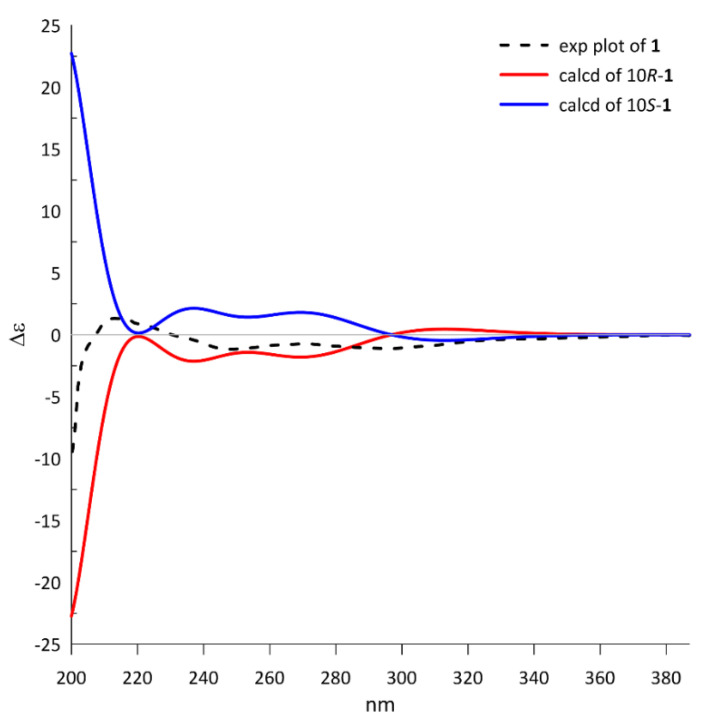
The calculated ECD spectra of 10*R*/10*S*-**1** at the PBE1PBE/tzvp level and the experimental plot of compound **1**.

Because only one chiral center was detected in **1**, the absolute configuration could be directly determined by comparing the theoretical ECD spectrum with the experimental plot. The calculated ECD spectrum of 10*R***-1** at the PBE1PBE/tzvp level exhibited an excellent fit to the experimental plot (Figure 3), assigning the 10*R* configuration.

Eurothiocin D (**2**), obtained as a colorless oil, showed a similar 1D NMR spectrum (Table 2) to that of eurothiocin A. The main differences observed were a series of additional proton signals from *δ*_H_ 3.30 to 5.20 including an anomeric oxymethine at *δ*_H_ 5.20 and six additional carbon resonances from *δ*_C_ 61 to 94, suggesting that a glucoside moiety should be constructed in compound **2**. The COSY correlations of H-1′/H-2′/H-3′/H-4′/H-5′/H_2_-6′ confirmed the existence of the glucoside; meanwhile, the HMBC correlations (Figure 2) from H-1 to C-11 further revealed the location of the glucoside at C-11.

**Figure 4:**
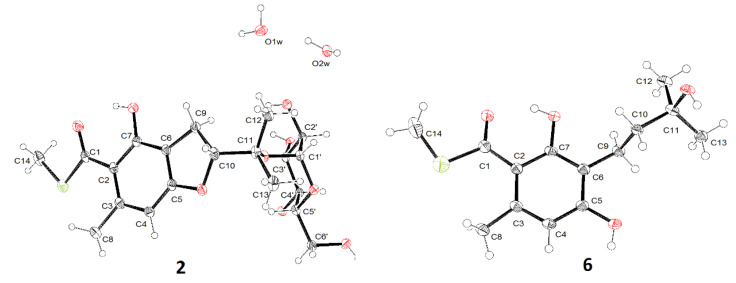
X-ray diffractions of compounds **2** and **6**.

The deshielded anomeric proton observed at *δ*_H_ 5.20 combined with the small ^3^*J*_1′,2′_ (3.8 Hz) indicated an *α*-glucopyranosyl linkage. Finally, the crystal of compound **2** was obtained and the X-ray diffraction analysis confirmed a *α*-d-glucopyranosyl unit at C-11 (Figure 4).

Eurothiocin E (**3**) was isolated as a colorless oil and gave the molecular formula C_14_H_17_O_3_S deduced by the quasi molecular ion peak at *m/z* 265.0900 [M – H]^−^ as well as the ^34^S-containing characteristic isotope ion peak at *m/z* 267.0879 [M – H + 2]^−^ in a 20:1 ratio with the ^32^S-containing ion peak from the HRESIMS spectrum. The ^1^H NMR spectrum (Table 1) displayed the signals of a chelating proton at *δ*_H_ 12.16 (7-OH), an aromatic proton at *δ*_H_ 6.22 (H-4), an intercoupled system composed of an olefin proton (*δ*_H_ 6.22), a methylene (*δ*_H_ 3.40) and four methyls at *δ*_H_ 2.63 (Me-8), 2.45 (Me-14), 1.81 (Me-13), and 1.74 (Me-12). The ^13^C NMR spectrum exhibited 14 signals containing eight olefinic carbons, a carboxyl carbon and five *sp^3^* hybridized carbons. Comprehensive analysis of the 1D NMR data suggested the existence of a penta-substituted benzene ring and an exocyclic *tri*-substituted double bond in compound **3**. 

The COSY correlation (Figure 2) of H_2_-9/H-10 combined with the HMBC cross-peaks from H_3_-12/H_3_-13 to C-10/C-11 constructed an isopentenyl unit (C-9 to C-13), which was connected to C-6 of the benzene ring based on the correlations from H_2_-9 to C-5/C-6/C-7. The locations of the carbonyl at C-1 and the chelating hydroxyl groups were evaluated by the HMBC correlations from 7-OH to C-2, C-6 and C-7. A similar methyl thioester moiety to that of eurothiocin C/D was deduced by the correlations from H_3_-14 to C-1 and the characteristic chemical shifts of C-1 (*δ*_C_ 198.2). All of the above evidence indicated that compound **3** was a ring-opening precursor of eurothiocin A. Finally, the HMBC correlation (Figure 2) from H_3_-8 to C-2/C-3/C-4 and the deshielded chemical shift of C-5 (*δ*_C_ 158.9) revealed a methyl and a hydroxy group at C-8 and C-5, respectively. Thus, the gross structure of compound **3** was established to be an isopentenyl-substituted benzoate thioester derivative. 

Both eurothiocins F (**4**) and G (**5**) exhibited similar 1D NMR spectra (Table 2 and Table 3) to that of compound **3**, which suggested that they are analogues containing a similar isopentenyl-substituted benzoate thioester core. Their HRESIMS spectra exhibited ^34^S:^32^S (20:1) isotope ion peaks. On the one hand, the main differences in compound **4** were that the methyl group (*δ*_H_ 1.74) was absent and an additional hydroxymethyl group at *δ*_H_ 3.98/*δ*_C_ 67.7 was detected, which indicated that Me-12 was oxygenated. On the other hand, in the 1D NMR spectrum of compound **5**, the signals of the *tri*-substituted double bond (Δ^10^) were absent and the olefin proton was transferred to a methylene (*δ*_H_ 3.98/*δ*_C_ 67.7), suggesting that the Δ^10^ was hydrolyzed. Further analysis of 2D NMR spectra (Figure 2) constructed the structures of both compound **4** and **5**. The geometry of the Δ^10^ in compound **4** was deduced by the NOESY correlation (Figure S38) between H-10 and H_2_-12; meanwhile, the structure of compound **5** was confirmed by X-ray diffraction analysis (Figure 4).

Eurothiocin H (**6**) was obtained as a colorless powder, the molecular formula of which was deduced to be C_10_H_11_O_3_S based on the HRESIMS data exhibiting a quasi molecular ion peak at *m/z* 211.0435 [M – H]^−^ and the ^34^S-containing isotope ion peak at *m/z* 213.0412 [M – H + 2]^−^. The methyl thioester moiety was evaluated by the characteristic methyl signals at *δ*_H_ 2.46/*δ*_C_ 12.6 and the carbonyl carbon at *δ*_C_ 195.2. A set of meta-coupled aromatic protons at *δ*_H_ 6.24/*δ*_H_ 6.27 detected in the ^1^H NMR spectrum and six aromatic carbon signals in ^13^C NMR revealed a benzoate thioester core in **6**. The HMBC correlations from H_3_-8 to C-2/C-3/C-4, from H_3_-9 to C-1, from H_3_-10 to C-7 and from H-4/H-6 to C-2/C-5 confirmed the complete structure.

To the best of our knowledge, thioester moieties are the rarest in marine microorganisms and only 12 compounds have been discovered to date, of which seven are produced by bacteria (suncheonosides A–D [14]; nitrosporeusines A–B [15]; thiocoraline [16]), two are produced by cyanobacteria (largazole [17] and thiopalmyrone [18]) and the others were isolated from marine mollusk-derived fungi (Eurothiocins A–B [13] and N-((3R,4S)-4-methyl-2-oxotetrahydrothiophen-3-yl)acetamide [19]). This is the first report of thioester-containing NPs isolated from the deep-sea-derived fungus. In general, it is recognized that glutathione is a direct donor of the sulfur atom in sulfur-containing NPs from plants or bacteria [11]. However, how the sulfur is introduced in the biosynthesis of metabolites from fungi is still unknown. A further biosynthetic investigation will be carried out in the future to understand the biosynthesis of the thioester moieties in eurothiocins.

### 2.2. Bioassays and Molecular Docking

**Figure 5:**
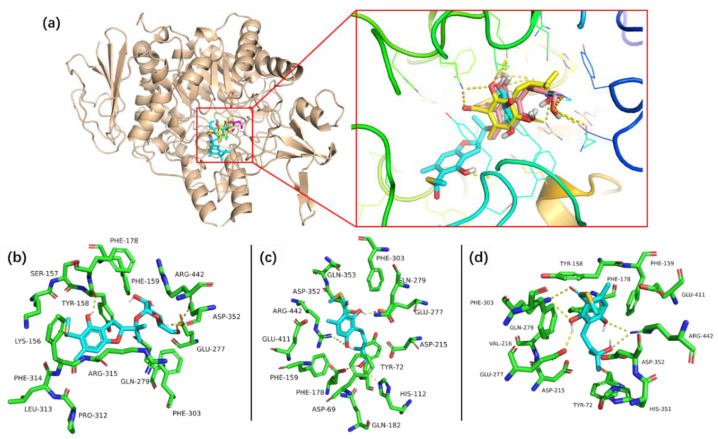
The complexes of compounds **1**, **4** and **5** binding in the active site of α-glucosidase (**a**) and the details of the interaction between compounds **1** (**b**), **4** (**c**), **5** (**d**) and the amino acid residue.

The previously reported bioactivity screening results indicated that eurothiocin A is a potential α-glucosidase inhibitor [13]. Thus, the in vitro α-glucosidase inhibitory activities of eurothiocins C–H (**1**–**6**) were evaluated (Table 4). Compound **2** exhibited the most significant inhibitory effect with an IC_50_ value of 5.4 μM, while compounds **4** and **5** showed moderate activities with IC_50_ values of 33.6 and 72.1 μM, respectively. Further, compounds **1**, **3** and **6** only displayed weak inhibitory effects at a concentration of 100 μM. The strong inhibitory effect of compound **2** might be contributed to by *α*-d-glucopyranosyl unit substitution at C-11, which could make it easy to interact with the enzyme. By comparing the compound with the structures and bioactivities of compounds **3**–**5**, it could be concluded that a hydrophilic terminal of the isopentenyl group at C-6 played an important role in inhibiting α-glucosidase. In order to predict the binding mechanism, a docking analysis of the reaction between α-glucosidase and the active compounds was performed through Autodock 4.2 (Figure 5). The protein crystallized structure of α-glucosidase from *Saccharomyces cerevisiae* was downloaded from the RSCB Protein Data Bank (pdb: 3A4A). The docking results indicated that compounds **2**, **4** and **5** bound at the same bioactive site, mainly containing the active residues Asp215, Val216, Gly217 or Ser218, which was reported by Yamamoto et al. previously [20]. Moreover, the hydrogen bonds between the 2'-OH of **2**/12-OH of **4**/11-OH of compound **5** and the residues in the site further demonstrated that the glucopyranosyl at C-11 or the hydrophilic terminal of the isopentenyl group might be the key bioactive function groups.

## 3. Materials and Methods

### 3.1. General Experimental Procedures

An Anton Paar MCP-500 (Anton Paar, Graz, Austria) and a Jasco 820 spectropolarimeter were used to measure the circular dichroism (ECD) and the UV spectra, respectively. ECD and UV spectra were recorded at the range of 200–400 nm under N_2_ gas production. IR spectra were recorded on a Shimadzu IR Affinity-1 spectrophotometer. All the NMR spectra were measured on a 600 MHz Bruker Avance-III HD spectrometer and the tetramethylsilane was used as an internal standard. HR-ESI-MS was measured on a Bruker maXis high-resolution mass spectrometer. A Shimadzu LC-20 AT (equipped with an SPD-M20A PDA detector) was used for the preparative separations. The ACE 5 PFP-C_18_ column (250 × 10.0 mm, 5 μm, 12 nm) was used for semi-preparative HPLC separation and the CHIRAL-MD (2)-RH column (250 × 10.0 mm, 5 μm) was used for chiral separation (Guangzhou FLM Scientific Instrument Co., Ltd, Guangzhou, China). The commercial silica gel (SiO_2_; 200–300 mesh; Qingdao Marine Chemical Plant, Qingdao, China) and Sephadex LH-20 gel (GE Healthcare Bio-Sciences ABSE-751, Uppsala, Sweden) were used for column chromatography. All solvents were of analytical grade (Guangzhou Chemical Regents Company, Ltd., Guangzhou, China). The natural sea salt was purchased from Guangdong Yueyan saltern. The *α*-glucosidase from *Saccharomyces cerevisiae* and *p*-nitrophenyl-α-d-glucopyranoside (*p*-NPG) used in bioassays were purchased from Sigma-Aldrich (St. Louis, MO, USA).

### 3.2. Fungal Material

The fungal strain FS688 was identified to be *Talaromyces indigoticus*, which was collected from deep-sea sediment in the South China Sea (118° 19.692′ N, 20° 38.982′ E; depth 2372 m) in September 2020. Fungal identification was carried out by morphological traits and ITS rDNA sequence analysis. The sequence data have been submitted to GenBank, under accession number OL774516. The strain was deposited at the Guangdong Provincial Key Laboratory of Microbial Culture Collection and Application, Institute of Microbiology, Guangdong Academy of Sciences. Working stocks were prepared on PDA (agar 4 g/L, potato 200 g/L, glucose 20 g/L, KH_2_PO_4_ 3 g/L, MgSO_4_•7H_2_O 1.5 g/L, vitamin B_1_ 10 mg/L, natural sea salt 15 g/L) slants stored at 4 °C.

### 3.3. Fermentation, Extraction, and Isolation

The grown mycelia were inoculated in 250 mL of PDB culture for 5 days in a 200 rpm rotary shaker at 28 °C. Then, the culture was transferred into the rice medium (12 Erlenmeyer flasks each containing 250 g rice and 400 mL H_2_O with 0.3% natural sea salt) and incubated at room temperature for another 30 days. After fermentation, the solid fermented substrate was extracted with methanol three times to obtain a dark brown oily residue (20.3 g), which was further subjected to silica gel column chromatography, eluting with petroleum ether/EtOAc in a linear gradient (9:1 to 1:9) to afford six fractions (Fr.1–Fr. 6). Compounds **3** (250 mg) and **7** (600 mg) were purified from Fr.1 by Sephdex-20 (eluting with 1:1 MeOH/CH_2_Cl_2_) and silica gel column (eluting with 20% petroleum ether/EtOAc), respectively. Fr.2 was subjected to repeated silica gel column chromatography, eluting with MeOH/CH_2_Cl_2_ to obtained crude **1**, which was further purified by HPLC equipped with a PFP-C_18_ column to obtain compounds **1** (7.2 mg, 80% MeOH/H_2_O, 2 mL/min, T_R_ = 12.5 min), **6** (8.0 mg, 80% MeOH/H_2_O, 2 mL/min, T_R_ = 10.5 min) and **5** (20 mg, 70% MeOH/H_2_O, 2 mL/min, T_R_ = 25.5 min). Fr.3 was subjected to repeated silica gel column chromatography, Sephdex-20 (eluting with 50% MeOH/CH_2_Cl_2_) followed by HPLC with an Ar-C_18_ column to obtain compound **4** (1.6 mg, 90% MeOH/H_2_O, 2 mL/min, T_R_ = 9.5 min). Fr. 6 was subjected to HPLC equipped with a PFP-C_18_ column to afford compound **2** (15.3 mg, 55% MeOH/H_2_O, 6 mL/min, T_R_ = 11.4 min).

Eurothiocin C (**1**): colorless oil; ${\left[\alpha \right]}_{D}^{25}$ + 3.8 (*c* 0. 1, MeOH); UV (MeOH) *λ*_max_ (log *ε*): 207 (4.17), 239 (3.71), and 295 (3.35) nm; IR (KBr): 2972, 2931, 2358, 1647, 1456, 1261, 1076, 1012, and 949 cm^−1^; ^1^H and ^13^C NMR data, Table 1. HRESIMS *m/z* 311.0964 [M − H]^−^ (calcd for C_15_H_19_O_5_S, 311.0959).

Eurothiocin D (**2**): colorless oil; ${\left[\alpha \right]}_{D}^{25}$ + 2.0 (*c* 0.1, MeOH); UV (MeOH) *λ*_max_ (log *ε*): 208 (4.17) 239 (3.72), and 295 (3.35) nm; IR (KBr): 3392, 2927, 2359, 1653, 1417, 1232, 1022, and 823 cm^−1^; ^1^H and ^13^C NMR data, Table 2. HRESIMS *m/z* 443.1391 [M − H]^−^ (calcd for C_20_H_27_O_9_S, 443.1381). 

Eurothiocin E (**3**): colorless oil; UV (MeOH) *λ*_max_ (log *ε*): 199 (4.15), 222 (3.68), and 283 (4.14) nm; IR (KBr): 3402, 2974, 2927, 2358, 1581, 1451, 1220, 1157, and 871 cm^−1^; ^1^H and ^13^C NMR data, Table 1; HRESIMS *m/z* 265.0900 [M − H]^−^ (calcd for C_14_H_17_O_3_S, 265.0904).

Eurothiocin F (**4**): colorless powder; UV (MeOH) 201 (4.15) and 296 (3.33) nm; IR (KBr): 3408, 2924, 2361, 1635, 1456, 1417, 1232, 1024, 1002, 981, and 820 cm^−1^; ^1^H and ^13^C NMR data, Table 1; HRESIMS *m/z* 281.0857, [M − H]^−^ (calcd for C_14_H_17_O_4_S, 281.0853). 

Eurothiocin G (**5**): colorless oil; UV (MeOH) *λ*_max_ (log *ε*): 201 (4.18) and 296 (3.33) nm; IR (KBr): 2979, 2926, 2358, 1522, 1418, 1227, 1101, and 974 cm^−1^; ^1^H and ^13^C NMR data, Table 3; HRESIMS *m/z* 307.0974 [M + Na]^+^ (calcd for C_28_H_34_NO_3_, 307.0975). 

Eurothiocin H (**6**): colorless powder; UV (MeOH) *λ*_max_ (log *ε*): 204 (4.18), 227 (3.55), and 276 (4.02) nm; IR (KBr): 3392, 2927, 2854, 2359, 2341, 1602, 1456, 1338, 1155, 1091, 1022, and 899 cm^−1^; ^1^H and ^13^C NMR data, Table 3. HRESIMS *m/z* 211.0435 [M − H]^−^ (calcd for C_10_H_11_O_3_S, 211.0434). 

Crystal diffraction data of compound **2**: Data were collected on an Agilent Xcalibur Nova single-crystal diffractometer using Cu Kα radiation. The crystal structure was refined by full-matrix least-squares calculation with the SHELXT. Crystallographic data have been deposited in the Cambridge Crystallographic Data Centre (deposition number: CCDC 2126903). Crystal data: C_20_H_32_O_11_S (M = 480.51); block crystal (0.1 × 0.08 × 0.06); space group P212121; unit cell dimensions a = 7.0695(1) Å, b = 7.9635(1) Å, c = 40.8016(6) Å, α = 90°, β = 90°, γ = 90°, V = 2297.05(6) Å^3^, and Z = 4; T = 100.0(1) K; ρ_cald_ = 1.389 mg/m^3^; absorption coefficient 1.765 mm^−1^; F(000) = 1024, a total of 11418 reflections were collected in the range 4.334° < θ < 74.356°, independent reflections 4535 [*R*(*int*) = 0.0269]; the number of data/parameters/restraints were 4535/0/318; goodness of fit on *F*^2^ = 0.957; final R indices [*I* > 2σ(I)] *R*_1_ = 0.0348 and ω*R*_2_ = 0.1111; R indices (all data) *R*_1_ = 0.0368 and ω*R*_2_ = 0.1129.

Crystal diffraction data of compound **5**: The conditions of data collection and program of refinement were the same as that of compound **2**. Crystallographic data have been deposited in the Cambridge Crystallographic Data Centre (deposition number: CCDC 2126902). Crystal data: C_14_H_20_O_4_S (M = 284.36); block crystal (0.20 × 0.15 × 0.10); space group P21/c; unit cell dimensions a = 6.1047(3) Å, b = 29.7526(14) Å, c = 8.0597(4) Å, α = 90°, β = 108.996(6)°, γ = 90°, V = 1384.17(13) Å^3^, and Z = 4; T = 100.0(1) K; ρ_cald_ = 1.365 mg/m^3^; absorption coefficient 2.155 mm^−1^; F(000) = 608, a total of 6484 reflections were collected in the range 5.449° < θ < 74.170°, independent reflections 2664 [*R*(*int*) = 0.0490]; the number of data/parameters/restraints were 2664/0/179; goodness of fit on *F*^2^ = 1.090; final R indices [I > 2σ(I)] *R*_1_ = 0.0819 and ω*R*_2_ = 0.2060; R indices (all data) *R*_1_ = 0.1036 and ω*R*_2_ = 0.2162.

### 3.4. Details of Quantum Chemical Calculations

Spartan’14 software (Wavefunction Inc.) and the Gaussian 09 program were used for the Merck molecular force field (MMFF) and DFT/TD-DFT calculations, respectively [21]. Conformers with an energy window lower the 10 kcal mol^−1^ from MMFF calculations were generated and re-optimized at the b3lyp/6-31+g(d,p) level, and the frequency calculations were carried out at the same level to estimate their relative thermal free energy (ΔG) at 298.15 K. Finally, conformers with the Boltzmann distribution over 5% were chosen for energy calculations at the b3lyp/6-311+g(d,p) level (rotatory strengths for a total of 20 excited states were calculated). The solvent effects were considered based on the self-consistent reaction field (SCRF) method with the polarizable continuum model (PCM). The final spectra were generated by the SpecDis program [22] using a Gaussian band shape with a 0.30 eV exponential half-width from dipole-length dipolar and rotational strengths. 

### 3.5. α-Glucosidase Inhibition Assay

The method used for the α-glucosidase inhibitory activity assay was based on previously reported literature with slight modification [23]. The pre-reaction mixture consisted of 130 μL of PBS (100 mM, pH 7.0), 30 μL of enzyme (1 U/mL) and 0.5 μL of the test compounds at different concentrations. After incubation at 37 °C for 10 min, 40 μL of *p*NPG was added and the mixture was further incubated at 37 °C for 15 min. Finally, the absorbance was measured at 405 nm on an automatic microplate reader. Acarbose was used as the positive control (IC_50_ = 317.2 µM). All experiments were carried out in triplicate.

### 3.6. Docking Analysis

The minimized structure of compound **2** was obtained from X-ray diffraction, while the preferred structures of compounds **4** and **5** were optimized by Spartan’14 software based on the MMFF. The energy grid maps for each atom type in the ligands as well as the electrostatic and de-solvation maps were calculated using the AutoGrid 4.2.6 program. The docking analysis was carried out in Autodock Tools package v1.5.4 (ADT, http://mgltools.scripps.edu/, 17 December 2021) based on a previously reported method. The docking pose was placed in a grid box of 90 × 90 × 90 Å^3^ (0.375 Å of grid spacing) with the protein at the center of the box [23]. The results were analyzed by ADT and the figures were prepared with PyMOL visualization tool (v1.7.4, Schrödinger, New York, NY, USA).

## Figures and Tables

**Table 1:** The ^1^H (600 MHz) and ^13^C NMR (150 MHz) data of compounds **1**, **3** and **4**.

Position	1 ^a^		3 ^a^		4 ^b^	
	*δ*_C_, Type	*δ*_H_	*δ*_C_, Type	*δ*_H_	*δ*_C_, Type	*δ*_H_
1	197.8, C		198.2, C		197.6, C	
2	116.3, C		115.4, C		115.6, C	
3	132.2, C		138.7, C		137.1, C	
4	135.8, C		111.7, CH	6.22, s	110.3, CH	6.23, s
5	156.9, C		158.9, C		158.9, C	
6	112.8, C		112.0, C		112.7, C	
7	153.2, C		160.5, C		159.0, C	
8	15.3, CH_3_	2.59, s	25.0, CH_3_	2.63, s	22.4, CH_3_	2.51, s
9	28.0, CH_2_	3.17, dd (15.6, 9.7)	22.1, CH_2_	3.40, d (7.1)	21.0, CH_2_	3.34, d (7.1)
		3.09, dd (15.6, 8.3)				
10	91.9, CH	4.78, dd (9.7, 8.3)	121.5, CH	5.25, tq (7.1, 1.4)	123.8, CH	5.48, brt (7.1)
11	72.0, C		135.0, C		134.0, C	
12	24.0, CH_3_	1.24, s	25.8, CH_3_	1.74,d (1.4)	67.7, CH_2_	3.89,d (1.4)
13	25.7, CH_3_	1.35, s	17.9, CH_3_	1.81, s	12.4, CH_3_	1.80, s
14	13.1, CH_3_	2.46, s	13.1, CH_3_	2.45, s	11.5, CH_3_	2.44, s
4-OMe	60.4, CH_3_	3.78, s				
7-OH		11.1, s		12.16, s		

^a^ recorded in chloroform-*d*; ^b^ recorded in methanol-*d*_4_.

**Table 2:** The ^1^H (600 MHz) and ^13^C NMR (150 MHz) data of compound **2**.

Position	2 ^a^				
	*δ*_C_, Type	*δ*_H_ (*J* in Hz)		*δ*_C_, Type	*δ*_H_ (*J* in Hz)
1	197.2, C		11	77.9, C	
2	119.8, C		12	20.5, CH_3_	1.30, s
3	137.8, C		13	21.3, CH_3_	1.33, s
4	103.3, CH	6.19, s	14	11.3, CH_3_	2.43, s
5	162.9, C		1′	93.2, CH	5.20, d (3.8)
6	110.7, C		2′	72.1, CH	3.35, dd (9.8, 3.8)
7	152.6, C		3′	73.6, CH	3.54, t (9.8)
8	20.0, CH_3_	2.32, s	4′	70.4, CH	3.30, m
9	27.7, CH_3_	3.11, brd (3.8)	5′	72.1, CH	3.73, m
		3.13, brd (2.4)	6′	61.2, CH_2_	3.69, m
10	89.2, CH	4.80, t (8.8)			

^a^ Recorded in methanol-*d*_4_.

**Table 3:** The ^1^H (600 MHz) and ^13^C NMR (150 MHz) data of compounds **5** and **6**.

Position	5 ^a^		6 ^b^		
	*δ*_C_, Multiplicities	*δ*_H_ (*J* in Hz)		*δ*_C_, Multiplicities	*δ*_H_ (*J* in Hz)
1	197.7, C		1	195.2, C	-
2	116.0, C		2	122.7, C	
3	136.5, C		3	137.8, C	
4	110.2, CH	6.23, s	4	109.1, CH	6.24, d (2.1)
5	156.9, C		5	158.3, C	
6	114.1, C		6	96.8, CH	6.27, d (2.1)
7	153.2, C		7	157.5, C	
8	22.0, CH_3_	2.48, s	8	19.1, CH_3_	2.24, s
9	17.5, CH_2_	2.65, m	9	12.6, CH_3_	2.46, s
			10	55.9, CH_3_	3.78, s
10	41.7, CH	1.63, m			
11	70.4, C				
12	27.6, CH_3_	1.24, s			
13	27.6, CH_3_	1.24, s			
14	11.5, CH_3_	2.44, s			

^a^ Recorded in methanol-*d*_4_. ^b^ Recorded in chloroform-*d*.

**Table 4:** α-Glucosidase inhibitory activities of compounds **1**–**6**.

Compounds	IC_50_ (μM)
**1**	>100
**2**	5.4
**3**	>100
**4**	33.6
**5**	72.1
**6**	>100
Acarbose	317.2

## Data Availability

Data are contained within the article or Supplementary Material.

## Supplementary Materials

The following supporting information can be downloaded at: https://www.mdpi.com/article/10.3390/md20010033/s1, Figures S1–S31. The NMR spectra of compounds **1**–**6**; Figures S32–S37. The HRESIMS spectra of compounds **1**–**6**; Figure S38. The key NOE correlation of compound **4**; Table S1. Energy analysis for the conformers of 10*R***-1**.
