# Supplementary material for: Thioester-Containing Benzoate Derivatives with α-Glucosidase Inhibitory Activity from the Deep-Sea-Derived Fungus Talaromyces indigoticus FS688
Source: Mar Drugs. 2021 Dec 29;20(1):33. doi: 10.3390/md20010033 (PMC8781869; doi:10.3390/md20010033)
Supplement: Supplementary file 1 [file marinedrugs-20-00033-s001.zip › marinedrugs-1538815-supplementary.pdf]

SUPPORTING INFORMATION

Thioester-containing benzoate derivatives with α-glucosidase inhibitory activity from the deep-sea-derived fungus *Talaromyces indigoticus* FS688

Mingqiong Li,[1] Saini Li,[1] Jinhua Hu,[1] Xiaoxia Gao,[2] Yanlin Wang,[3] Zhaoming Liu,[*,1] Weimin, Zhang[*,1]

[1]*State Key Laboratory of Applied Microbiology Southern China, Guangdong Provincial Key Laboratory of Microbial Culture Collection and Application, Institute of Microbiology, Guangdong Academy of Sciences, 100 Central Xianlie Road, Yuexiu District, Guangzhou 510070, China*;

[2]*College of Pharmacy, Guangdong Pharmaceutical University, Guangzhou 510006, China;*

[3]*Key Laboratory of Ocean and Marginal Sea Geology, South China Sea Institute of Oceanology, Innovation Academy of South China Sea Ecology and Environmental Engineering, Chinese Academy of Sciences, Guangzhou, China;*

Table of contents:

Figure S1. ^{1}H NMR spectrum of **1** in $CDCl_3$.
Figure S2. ^{13}C NMR spectrum of **1** in $CDCl_3$.
Figure S3. ^{1}H, ^{1}H-COSY spectrum of **1** in $CDCl_3$.
Figure S4. HSQC spectrum of **1** in $CDCl_3$.
Figure S5. HMBC spectrum of **1** in $CDCl_3$.
Figure S6. ^{1}H NMR spectrum of **2** in MeOD.
Figure S7. ^{13}C NMR spectrum of **2** in MeOD.
Figure S8. ^{1}H, ^{1}H-COSY spectrum of **2** in MeOD.
Figure S9. HSQC spectrum of **2** in MeOD.
Figure S10. HMBC spectrum of **2** in MeOD.
Figure S11. ^{1}H NMR spectrum of **3** in $CDCl_3$.
Figure S12. ^{13}C NMR spectrum of **3** in $CDCl_3$.
Figure S13. ^{1}H, ^{1}H-COSY spectrum of **3** in $CDCl_3$.
Figure S14. HSQC spectrum of **3** in $CDCl_3$.
Figure S15. HMBC spectrum of **3** in $CDCl_3$.
Figure S16. ^{1}H NMR spectrum of **4** in MeOD.
Figure S17. ^{13}C NMR spectrum of **4** in MeOD.
Figure S18. ^{1}H, ^{1}H-COSY spectrum of **4** in MeOD.
Figure S19. HSQC spectrum of **4** in MeOD.
Figure S20. HMBC spectrum of **4** in MeOD.
Figure S21. NOESY spectrum of **4** in MeOD.
Figure S22. ^{1}H NMR spectrum of **5** in MeOD.
Figure S23. ^{13}C NMR spectrum of **5** in MeOD.
Figure S24. ^{1}H, ^{1}H-COSY spectrum of **5** in MeOD.
Figure S25. HSQC spectrum of **5** in MeOD.
Figure S26. HMBC spectrum of **5** in MeOD.
Figure S27. ^{1}H NMR spectrum of **6** in $CDCl_3$.
Figure S28. ^{13}C NMR spectrum of **6** in $CDCl_3$.
Figure S29. ^{1}H, ^{1}H-COSY spectrum of **6** in $CDCl_3$.
Figure S30. HSQC spectrum of **6** in $CDCl_3$.
Figure S31. HMBC spectrum of **6** in $CDCl_3$.
Figure S32. HRESI TOF MS spectrum of **1**.
Figure S33. HRESI TOF MS spectrum of **2**.
Figure S34. HRESI TOF MS spectrum of **3**.
Figure S35. HRESI TOF MS spectrum of **4**.
Figure S36. HRESI TOF MS spectrum of **5**.
Figure S37. HRESI TOF MS spectrum of **6**.
Figure S38. The observed key NOE correlation between H-10 and H_2-12 in **4**.
Table S1. Energy analysis for the Conformers of 10*R*-**1**.

Figure S1. ^{1}H NMR spectrum of **1** in $CDCl_3$.

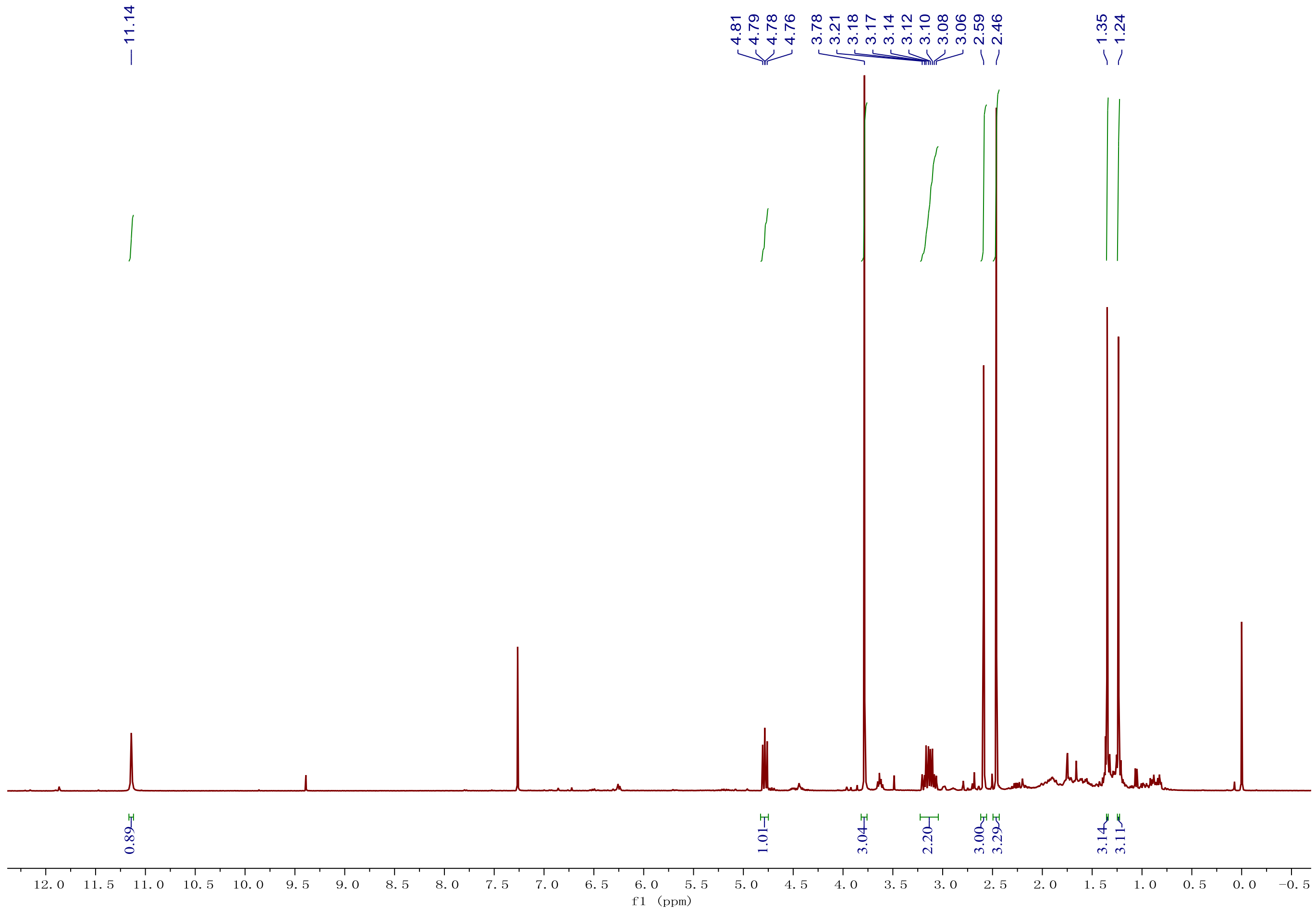

Figure S2. ^{13}C NMR spectrum of **1** in $CDCl_3$.

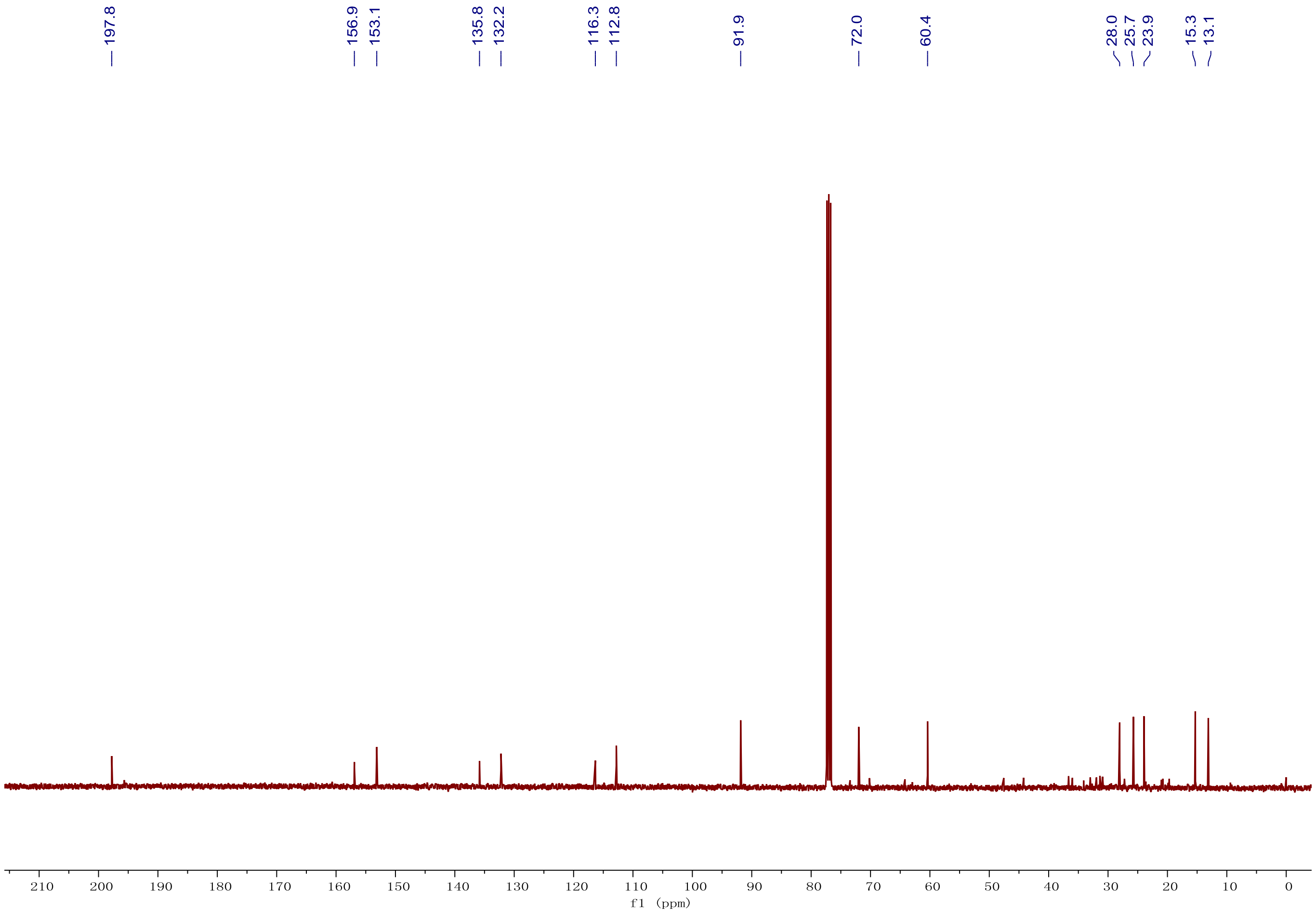

Figure S3. ^{1}H, ^{1}H-COSY spectrum of **1** in $CDCl_3$.

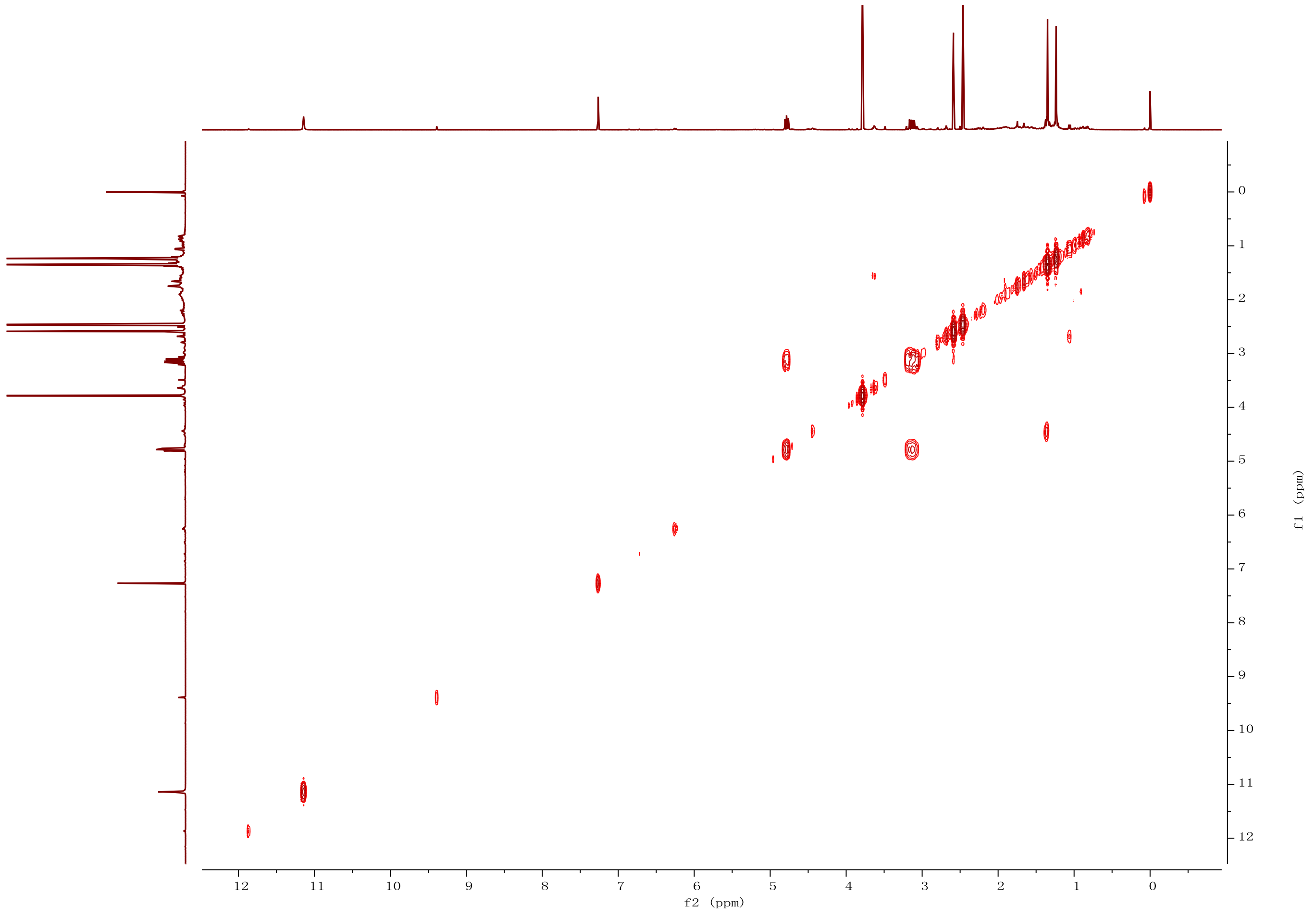

Figure S4. HSQC spectrum of **1** in $CDCl_3$.

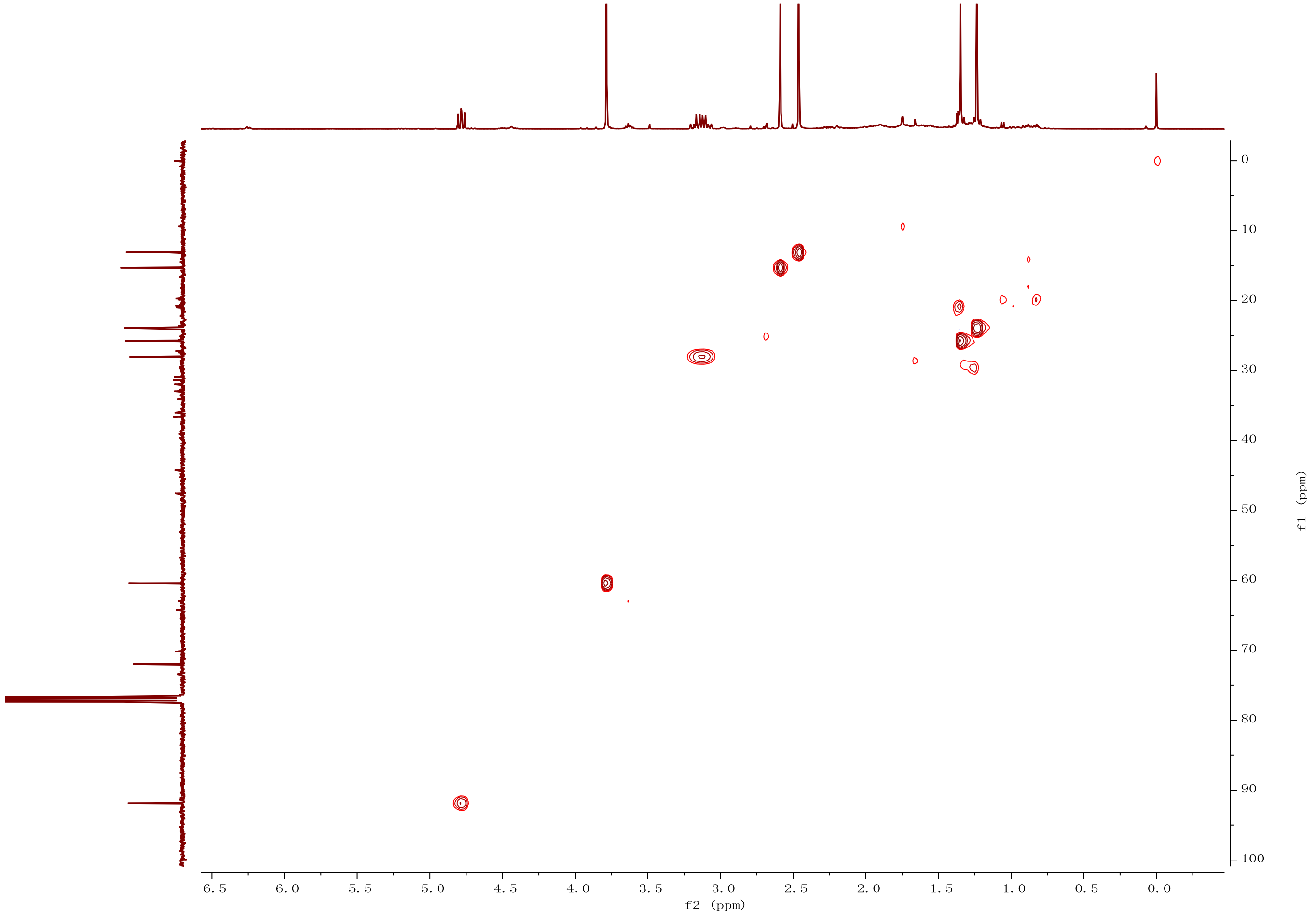

Figure S5. HMBC spectrum of **1** in $CDCl_3$.

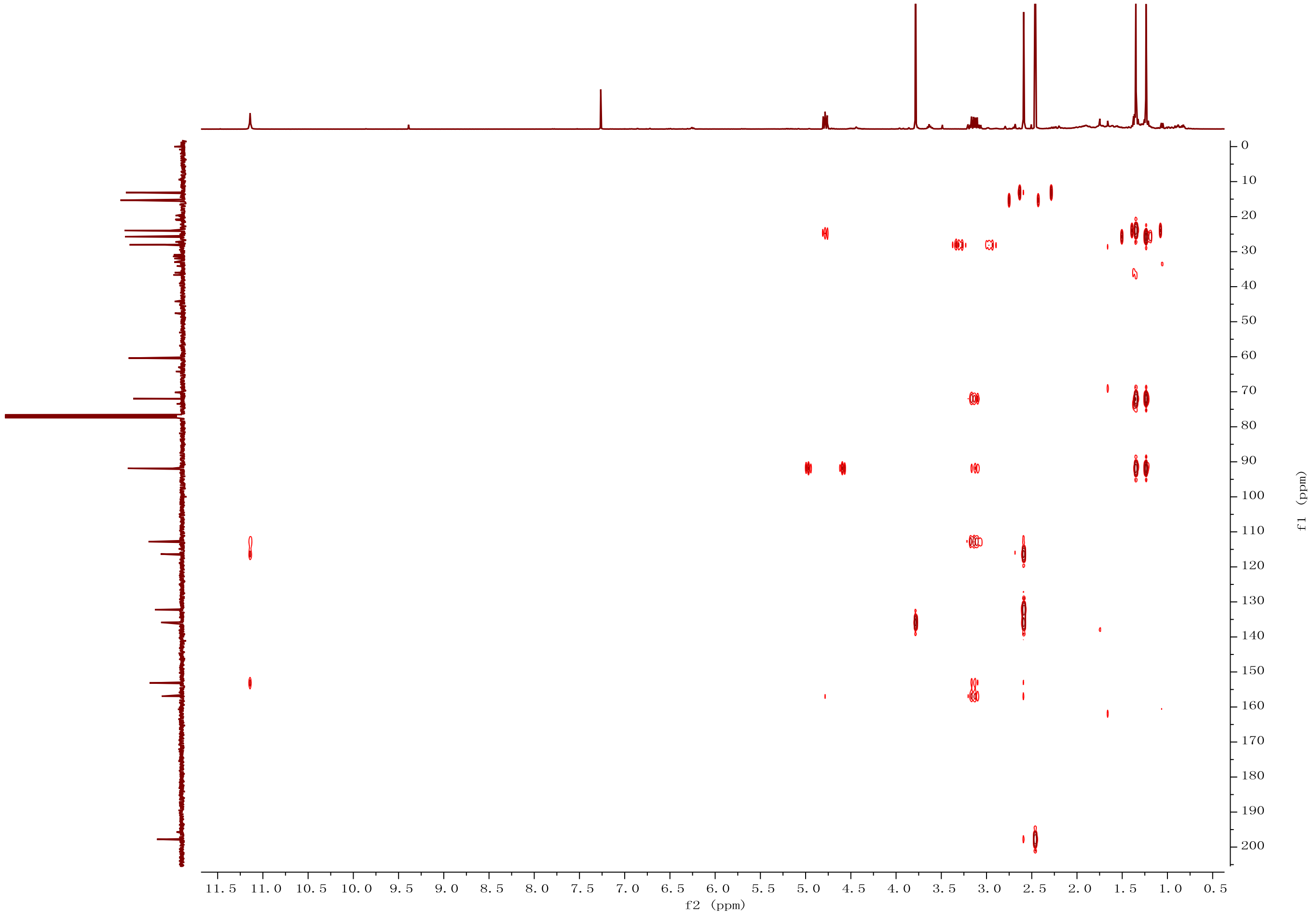

Figure S6. ^{1}H-NMR spectrum of **2** in MeOD.

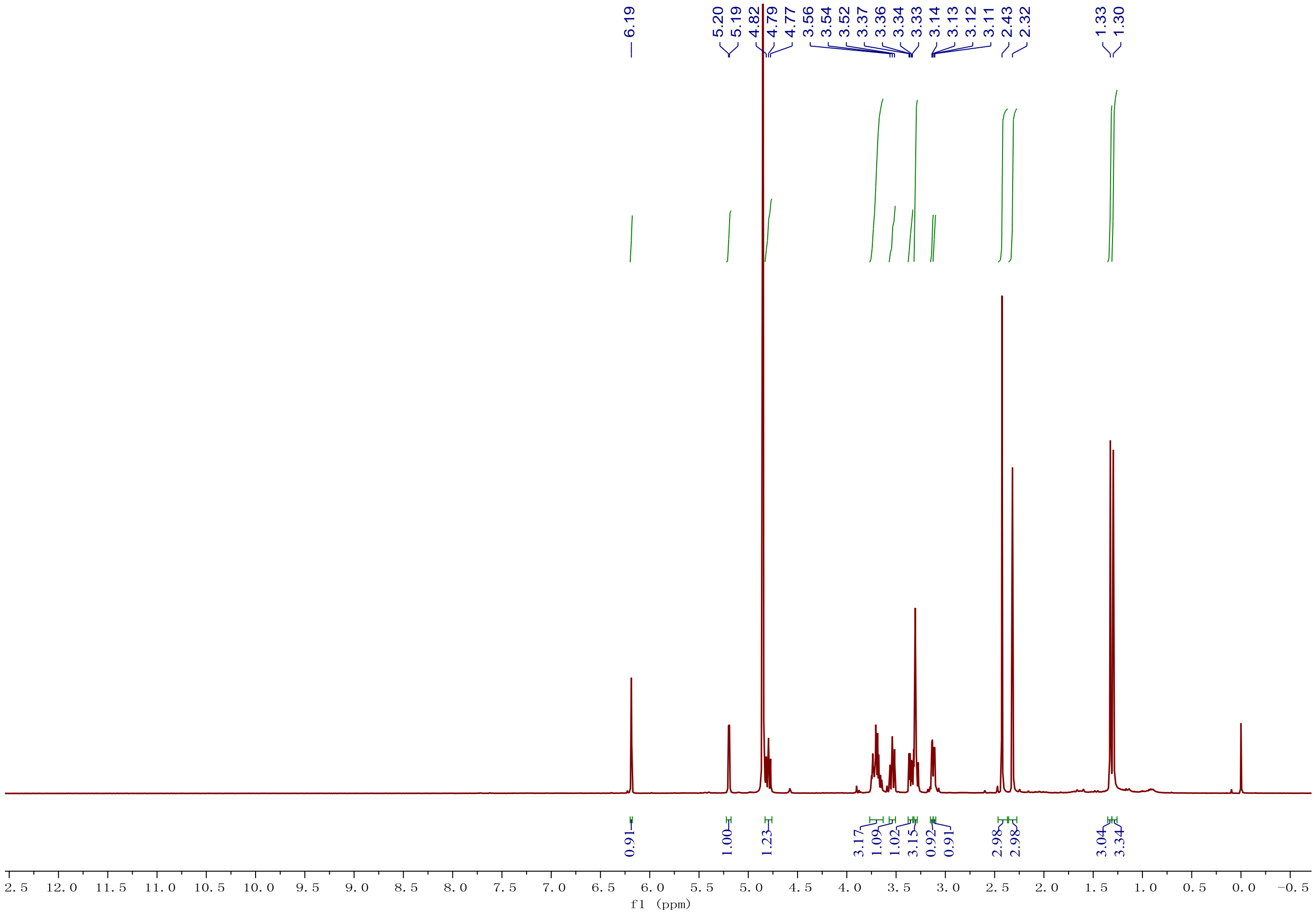

Figure S7. ^{13}C-NMR spectrum of **2** in MeOD.

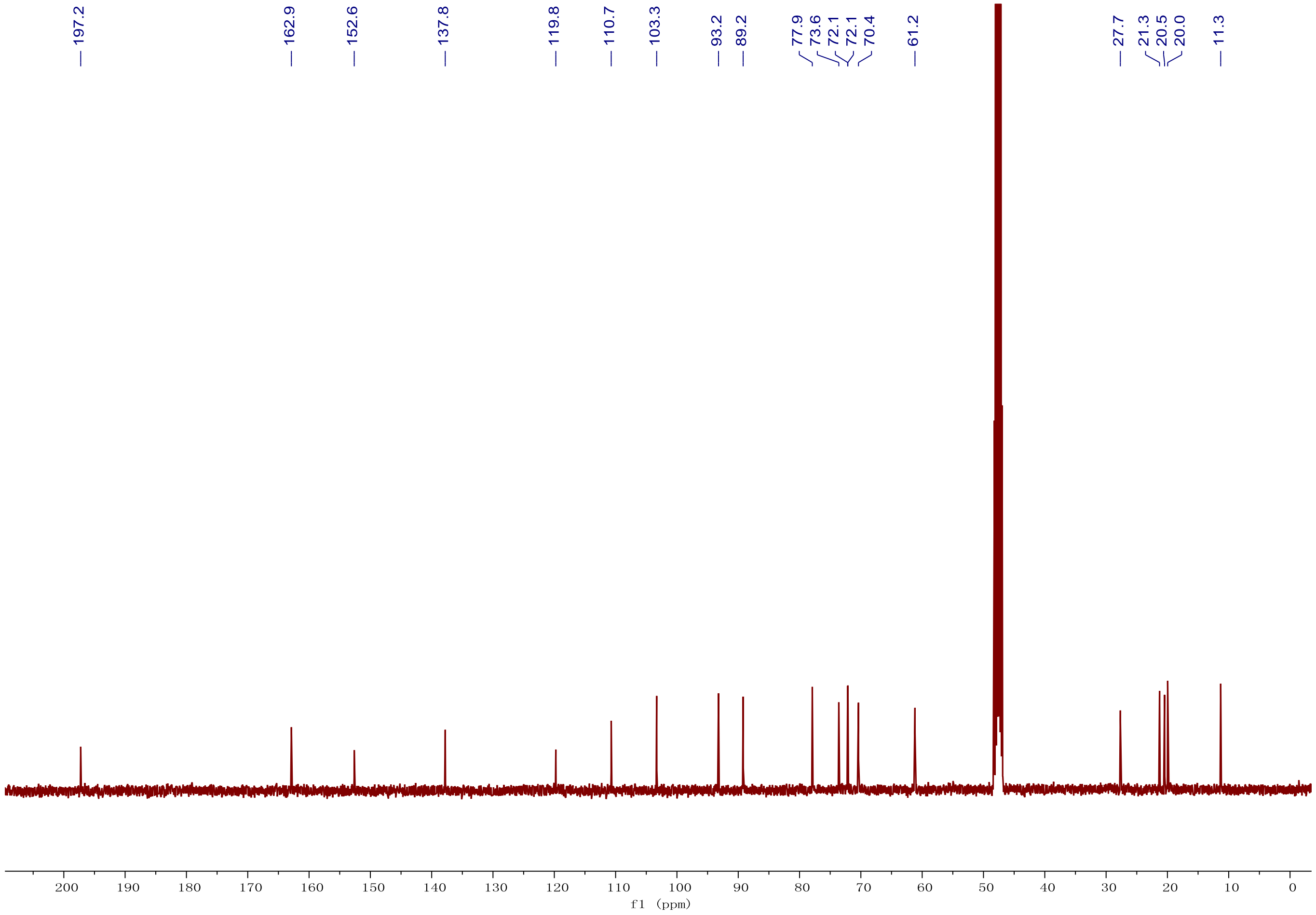

Figure S8. ^1H, ^1H-COSY spectrum of **2** in MeOD.

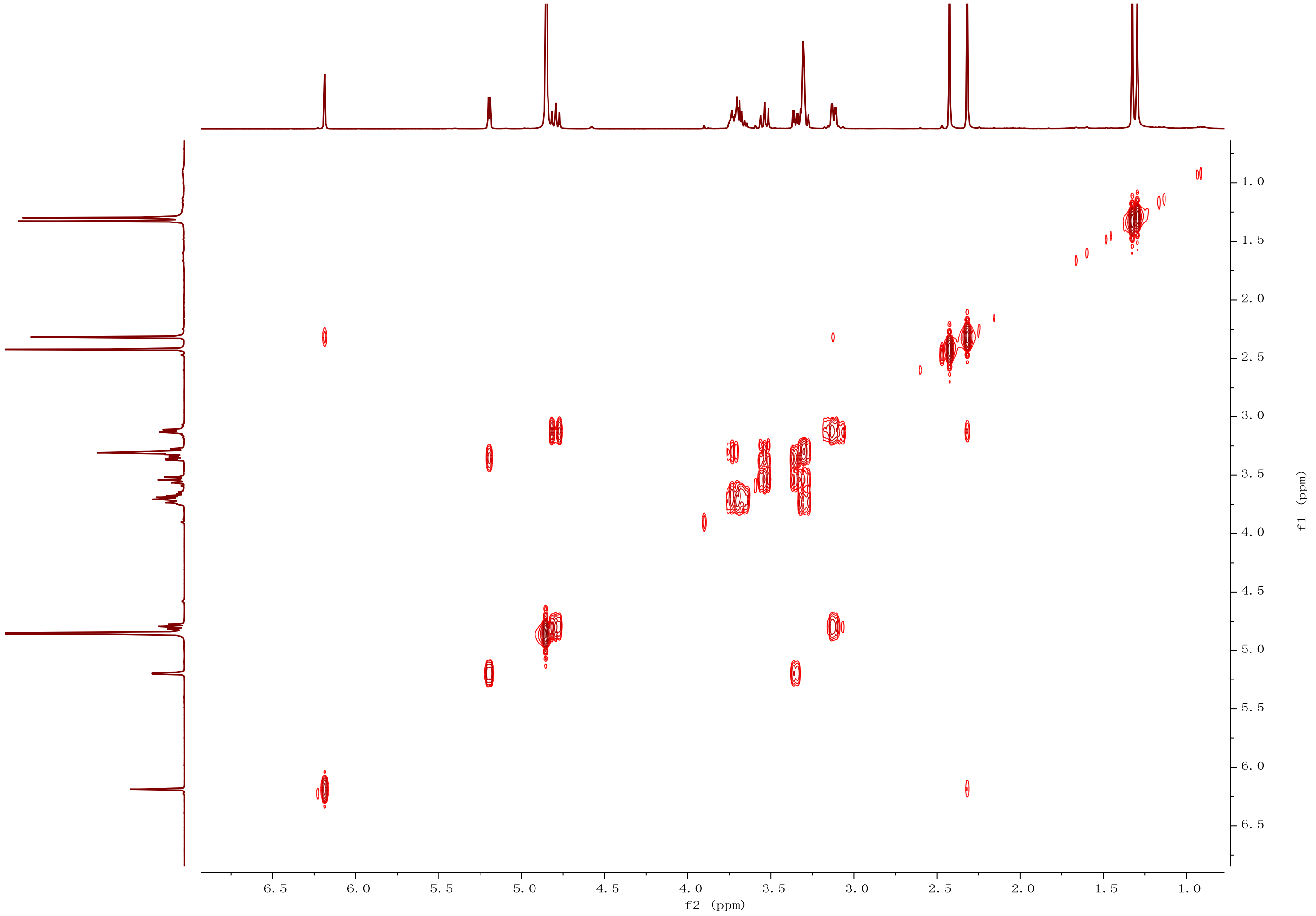

Figure S9. HSQC spectrum of **2** in MeOD.

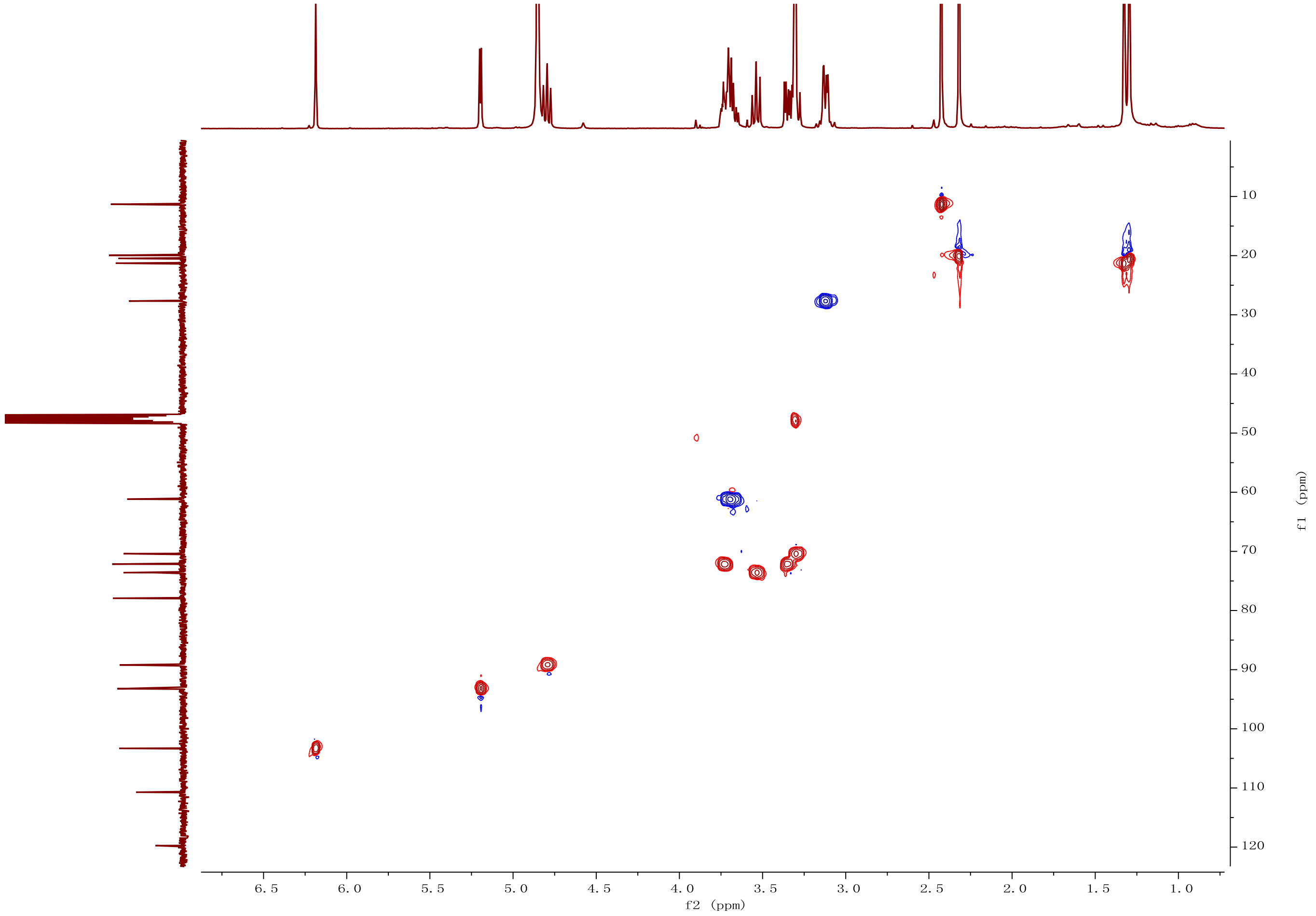

Figure S10. HMBC spectrum of **2** in MeOD.

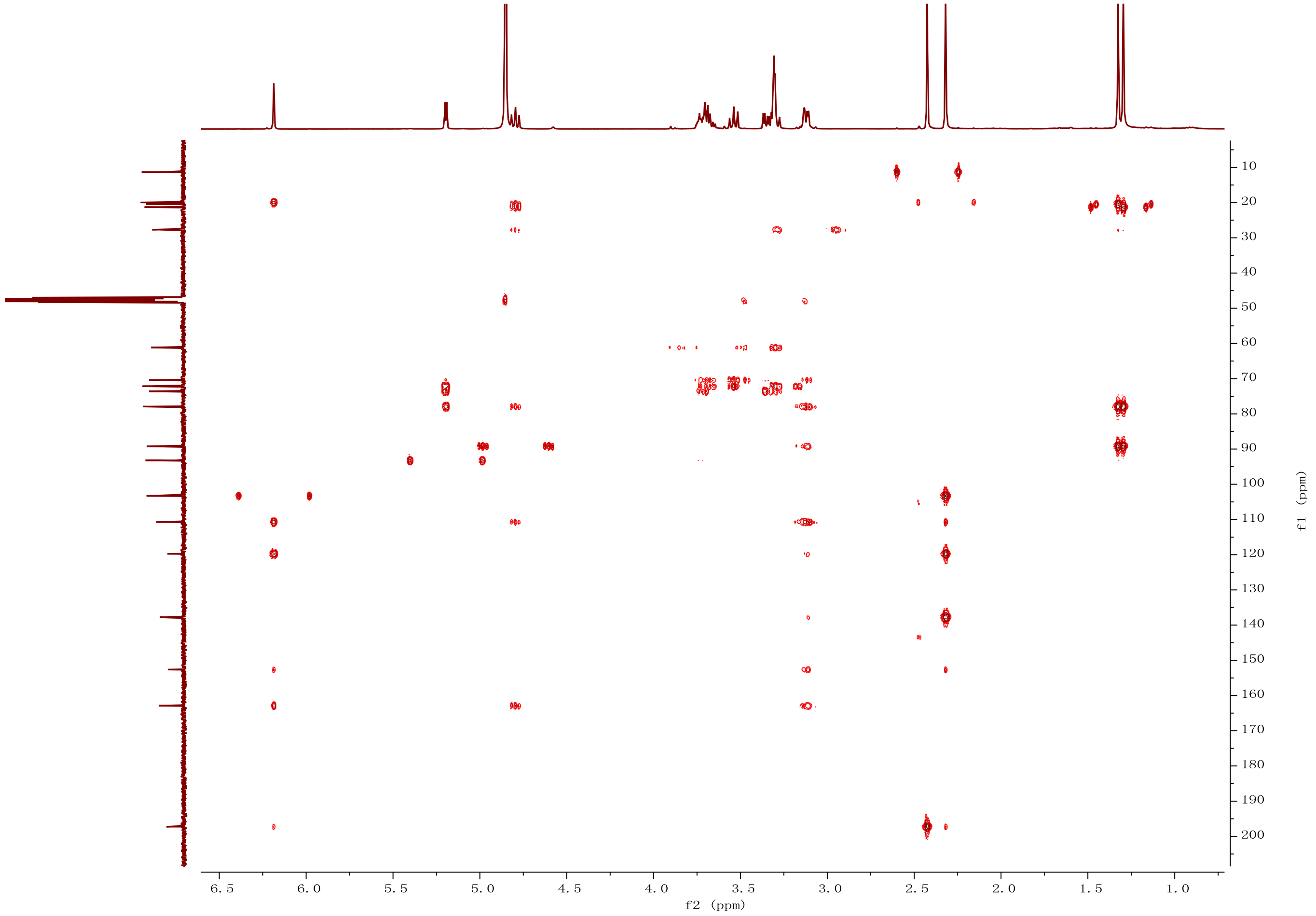

Figure S11. ^{1}H NMR spectrum of **3** in $CDCl_3$.

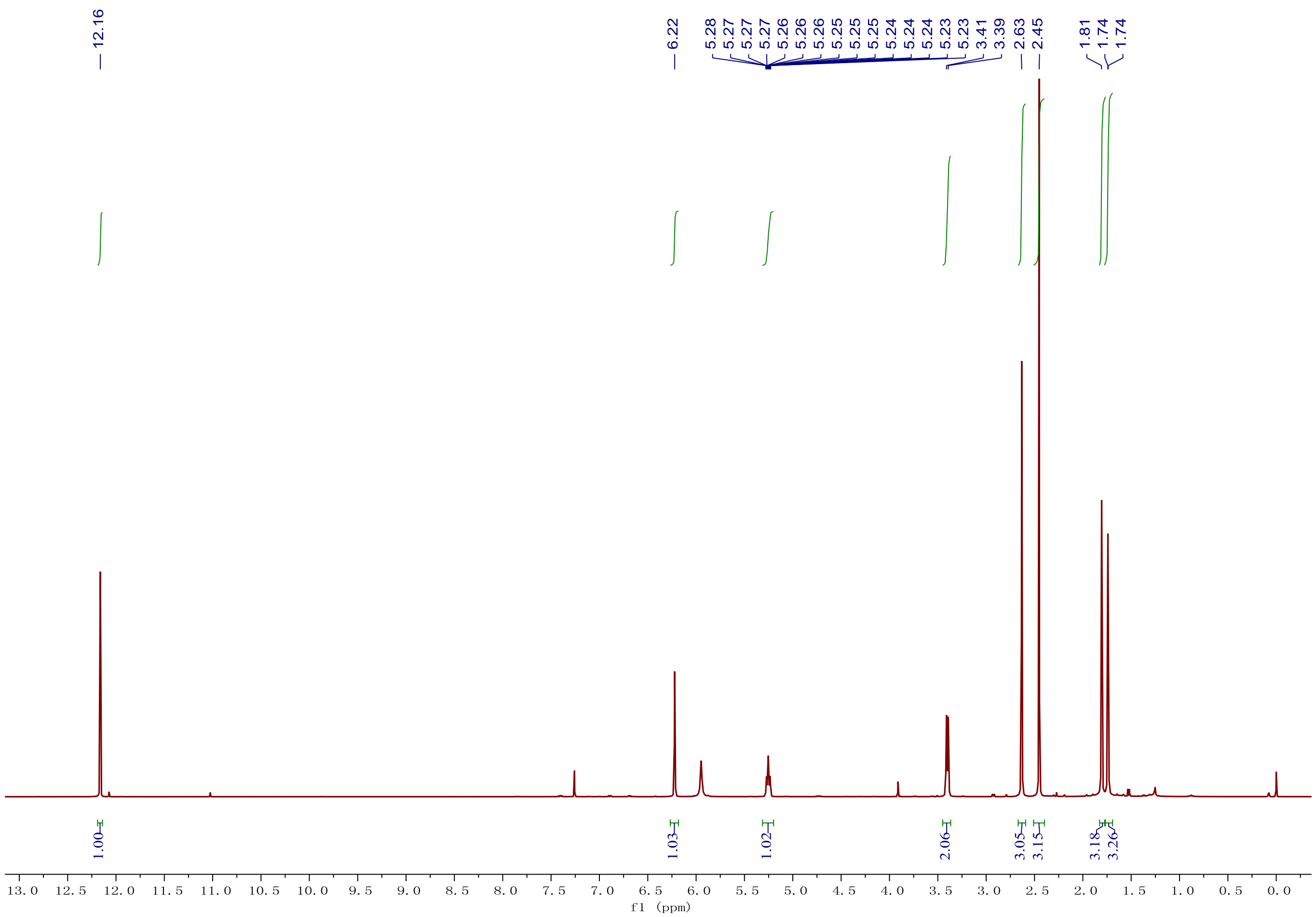

Figure S12. ^{13}C NMR spectrum of **3** in $CDCl_3$.

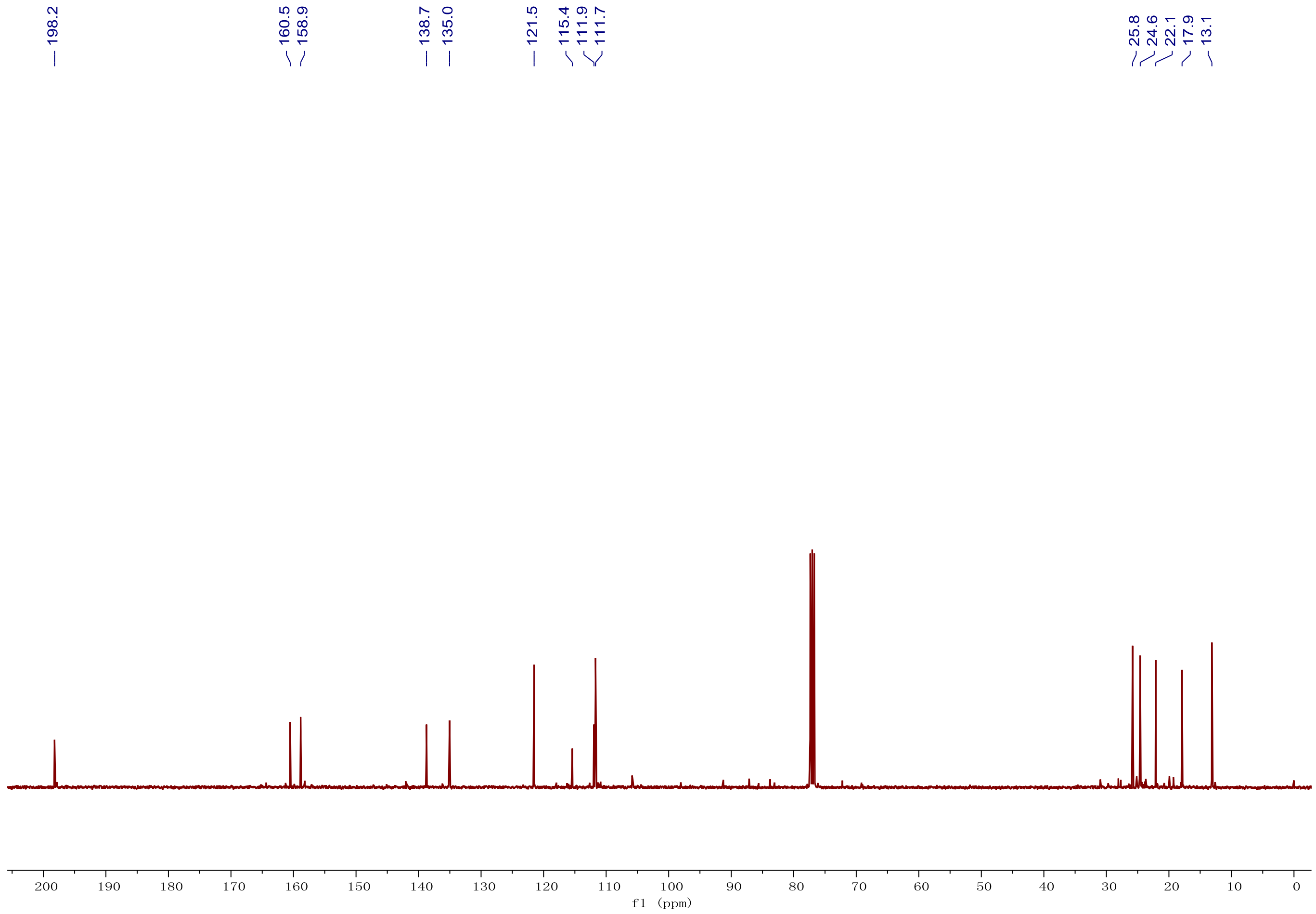

Figure S13. ^{1}H-^{1}H COSY spectrum of **3** in $CDCl_3$.

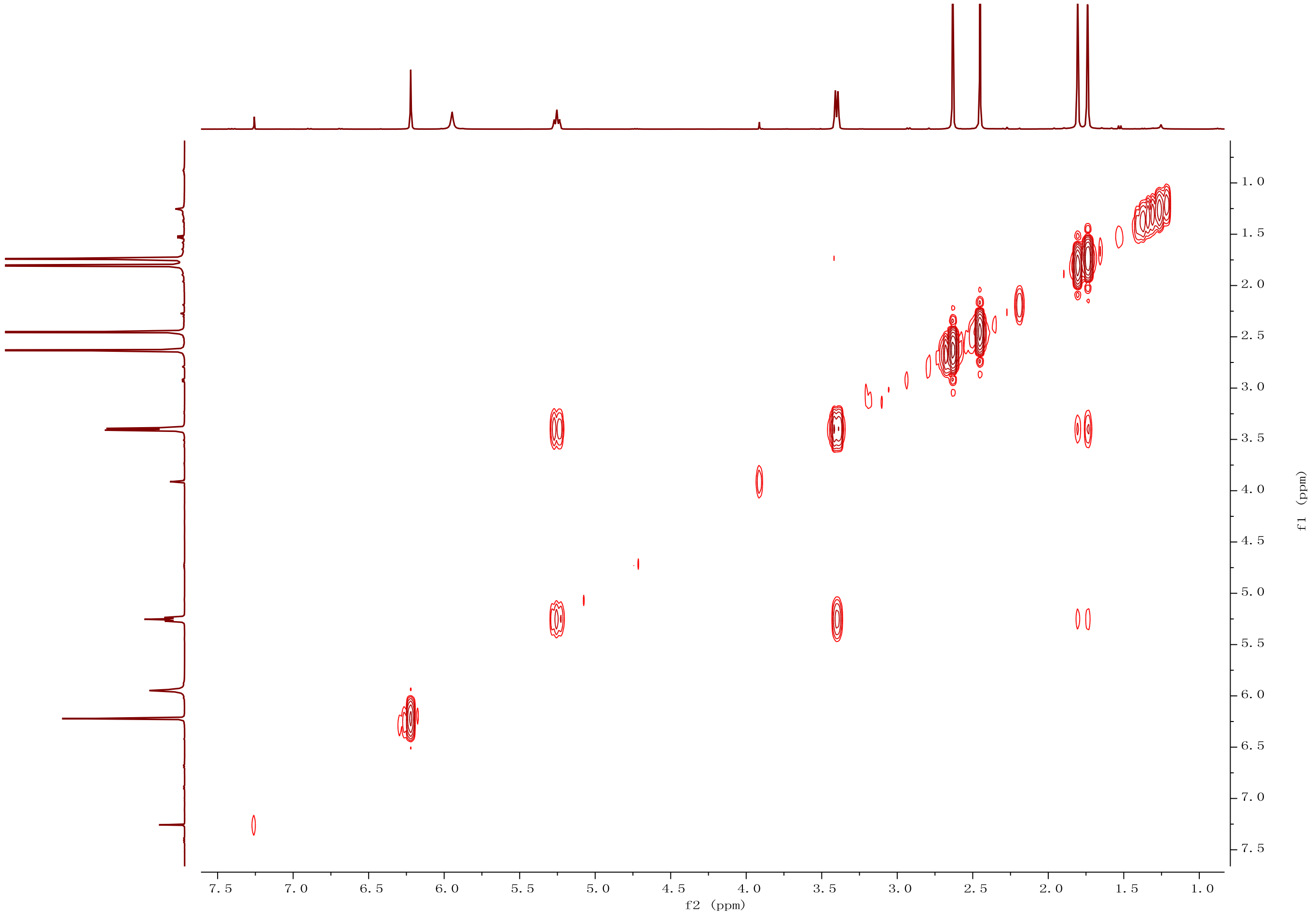

Figure S14. HSQC spectrum of **3** in $CDCl_3$.

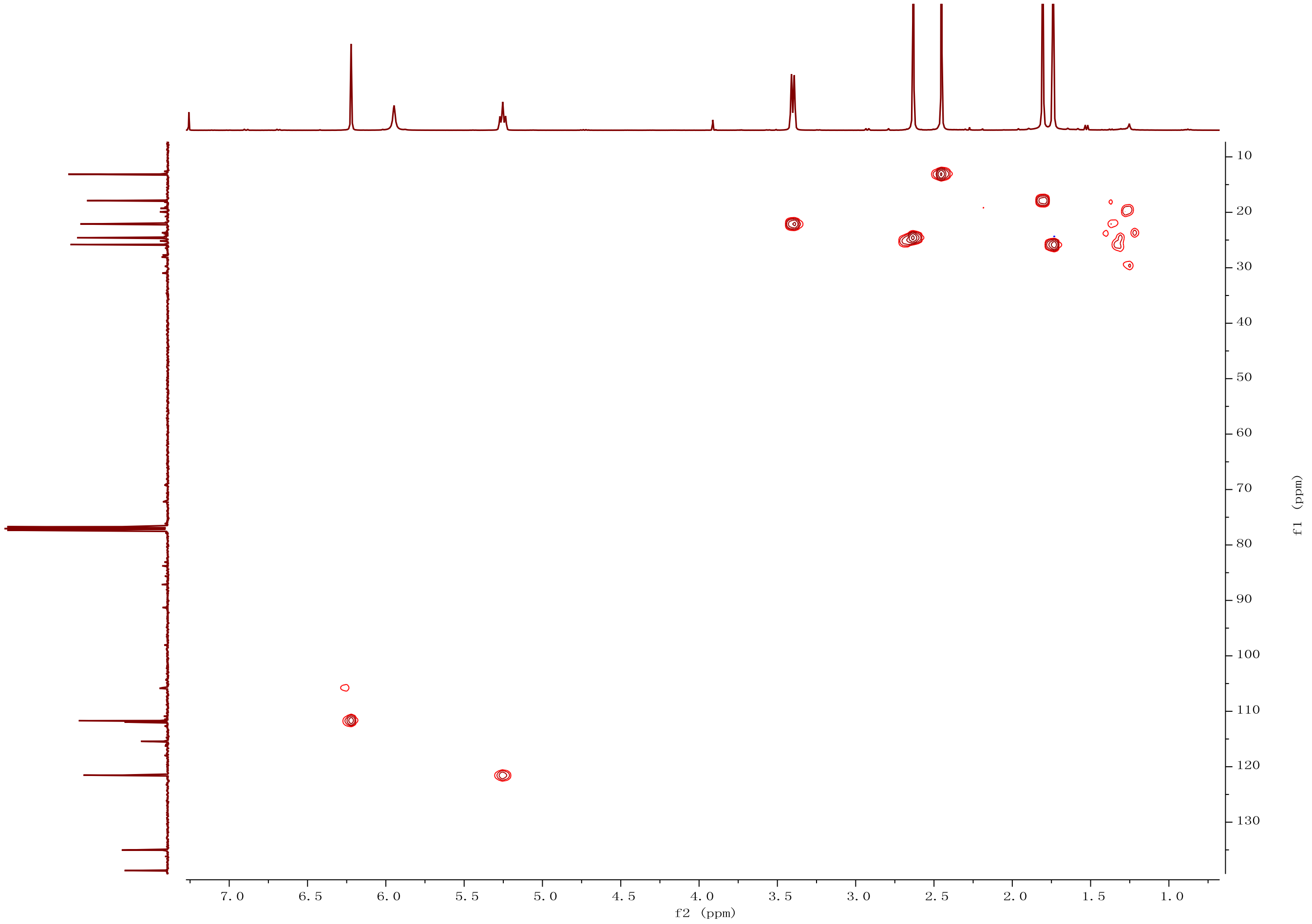

Figure S15. HMBC spectrum of **3** in $CDCl_3$.

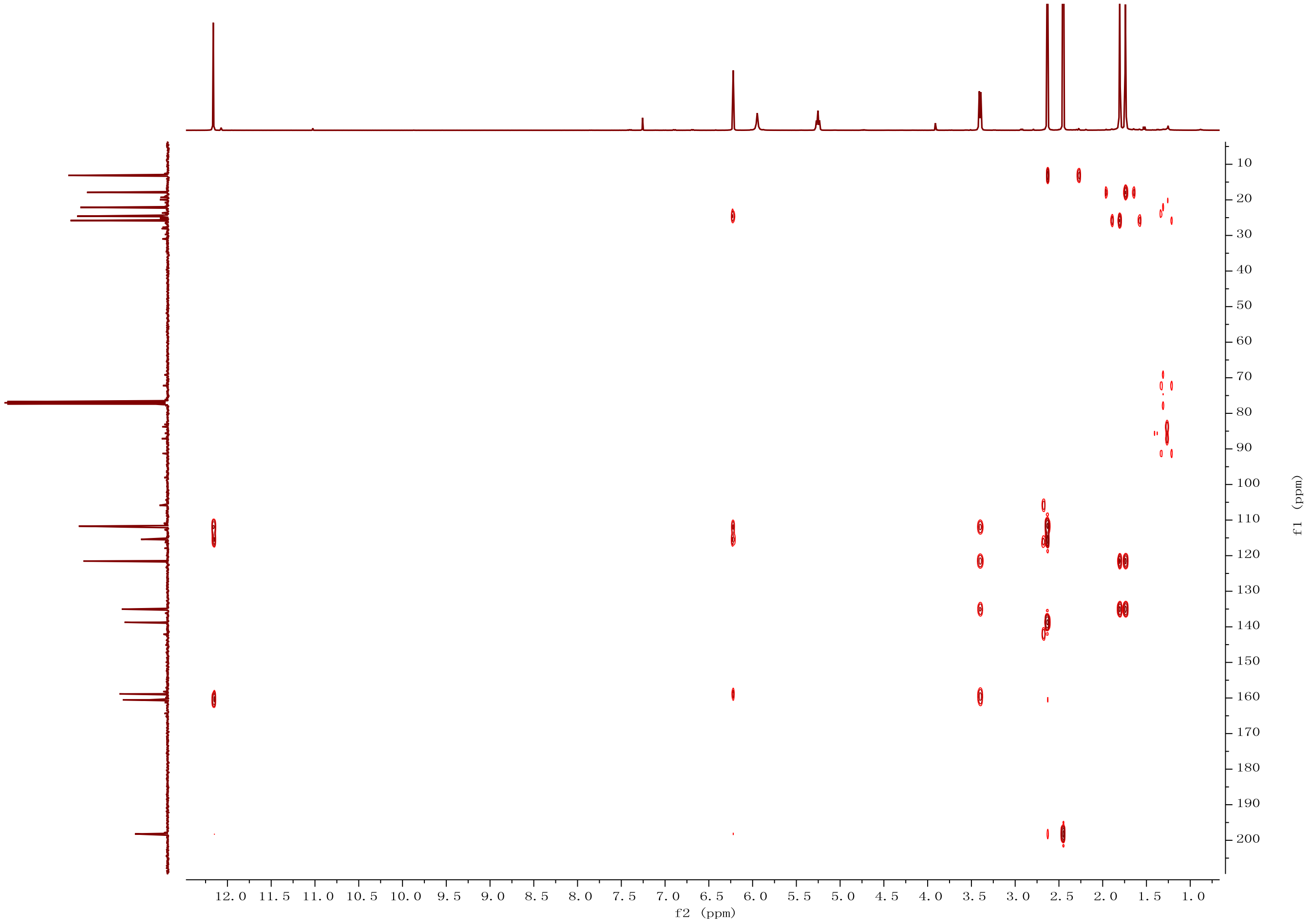

Figure S16. ^{1}H NMR spectrum of **4** in MeOD.

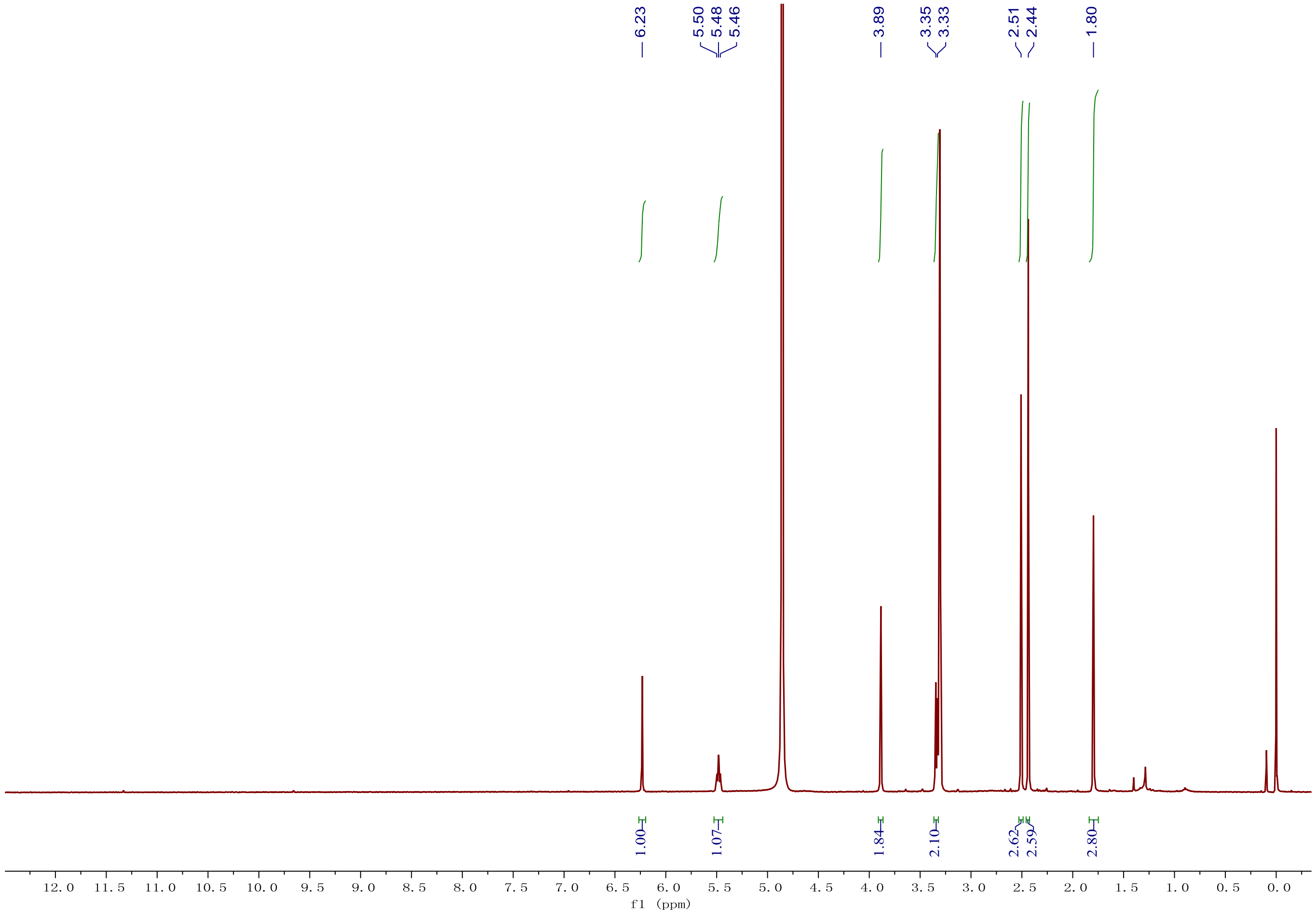

Figure S17. ^{13}C NMR spectrum of **4** in MeOD.

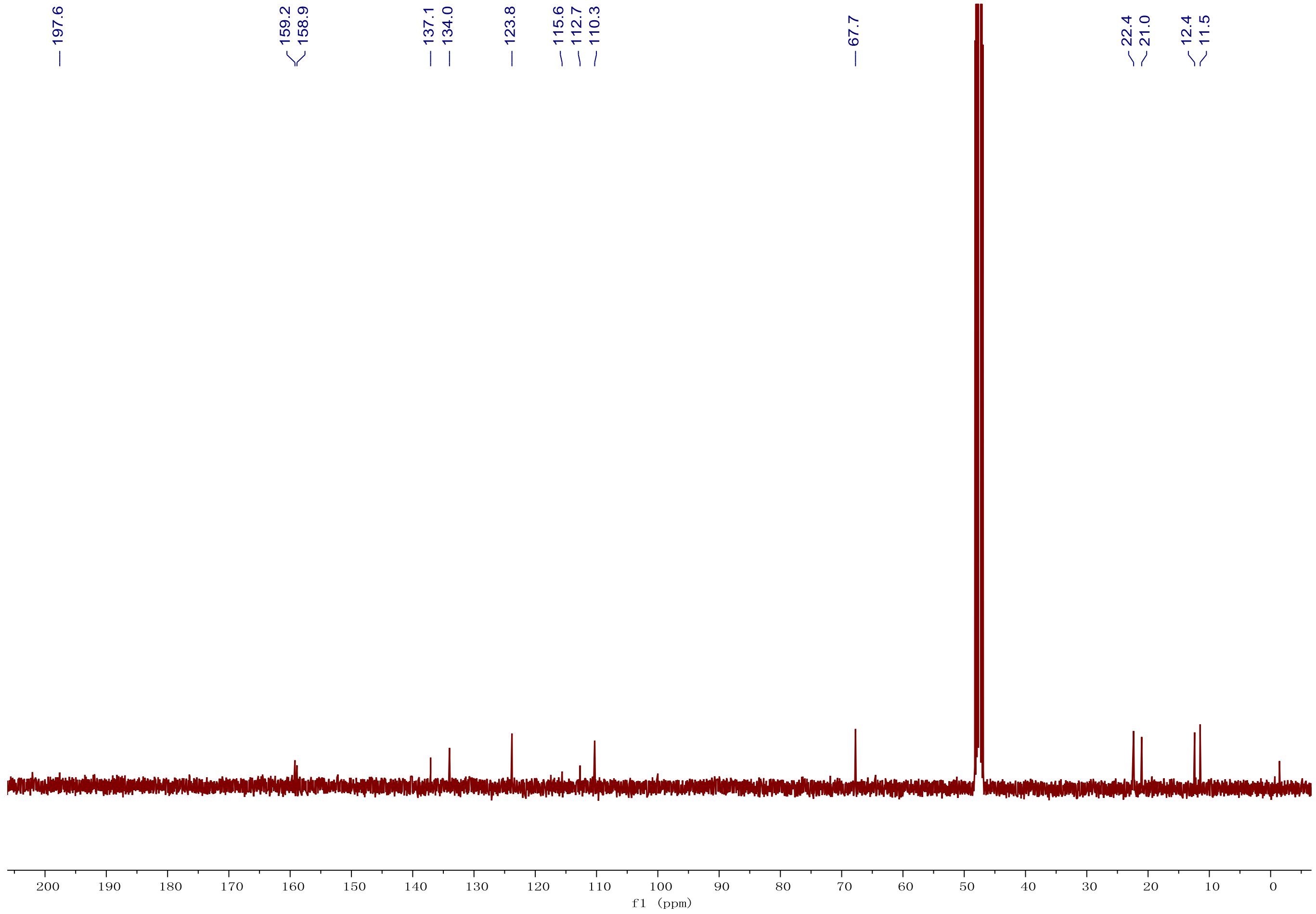

Figure S18. ^{1}H-^{1}H COSY spectrum of **4** in MeOD.

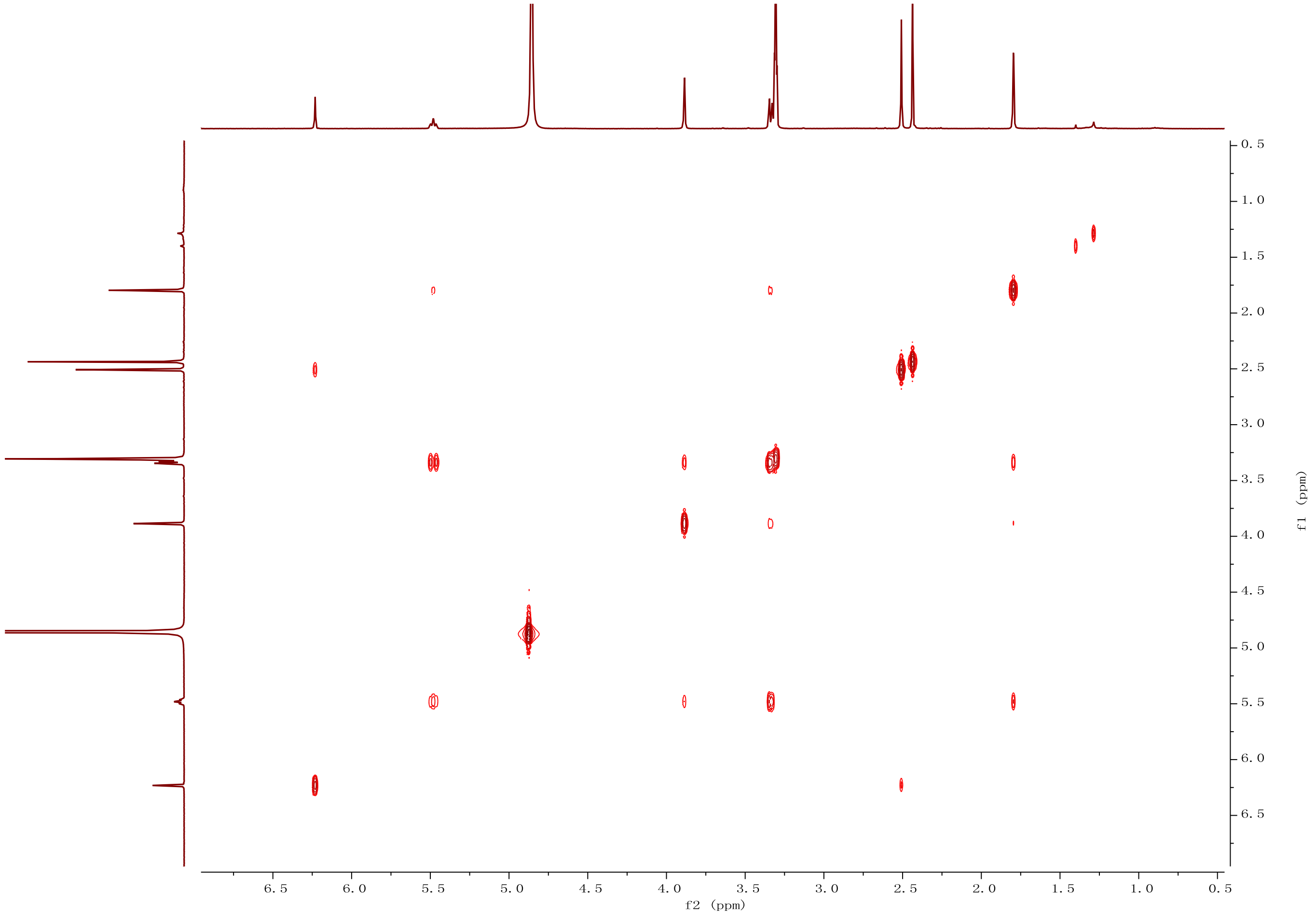

Figure S19. HSQC spectrum of **4** in MeOD.

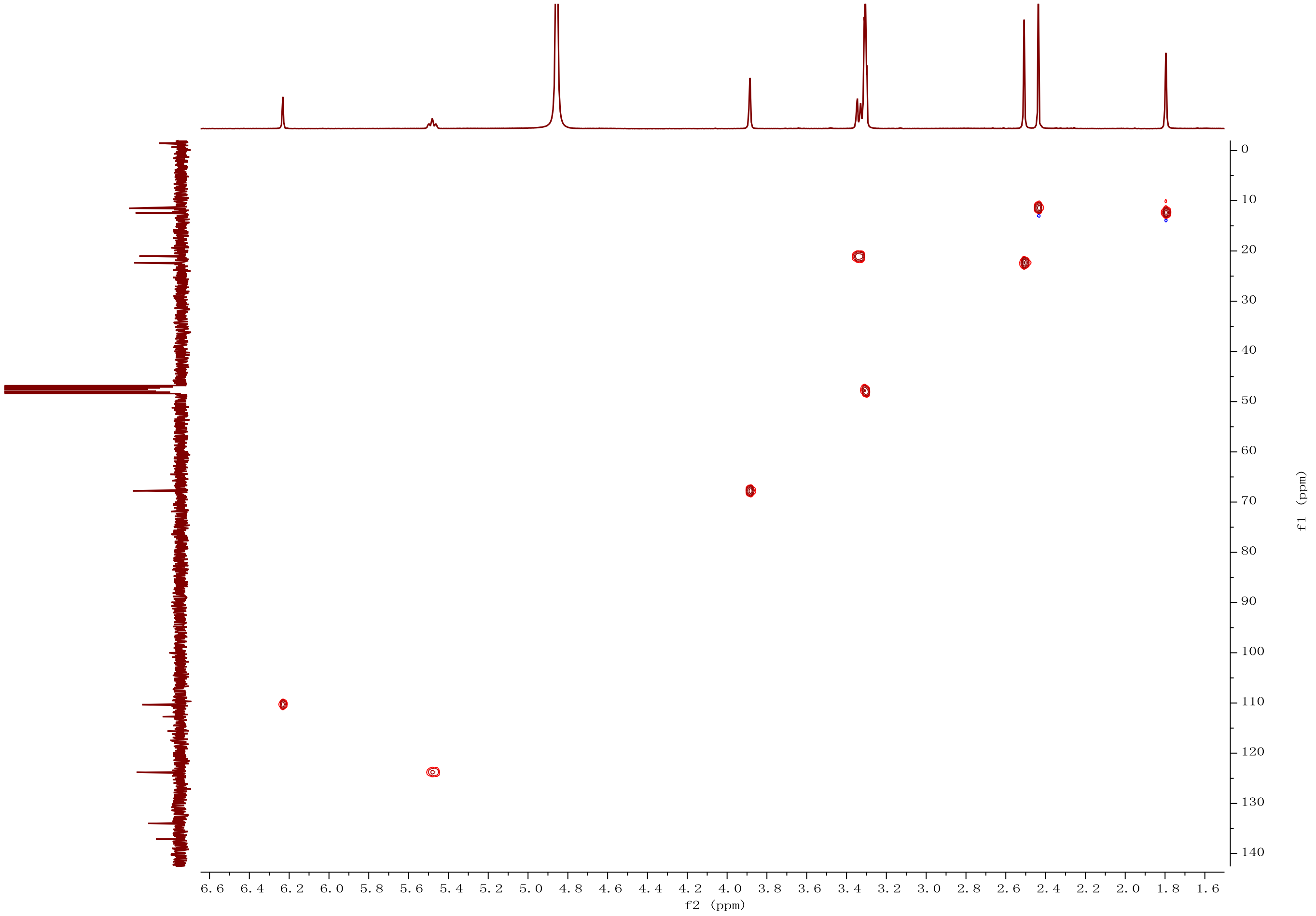

Figure S20. HMBC spectrum of **4** in MeOD.

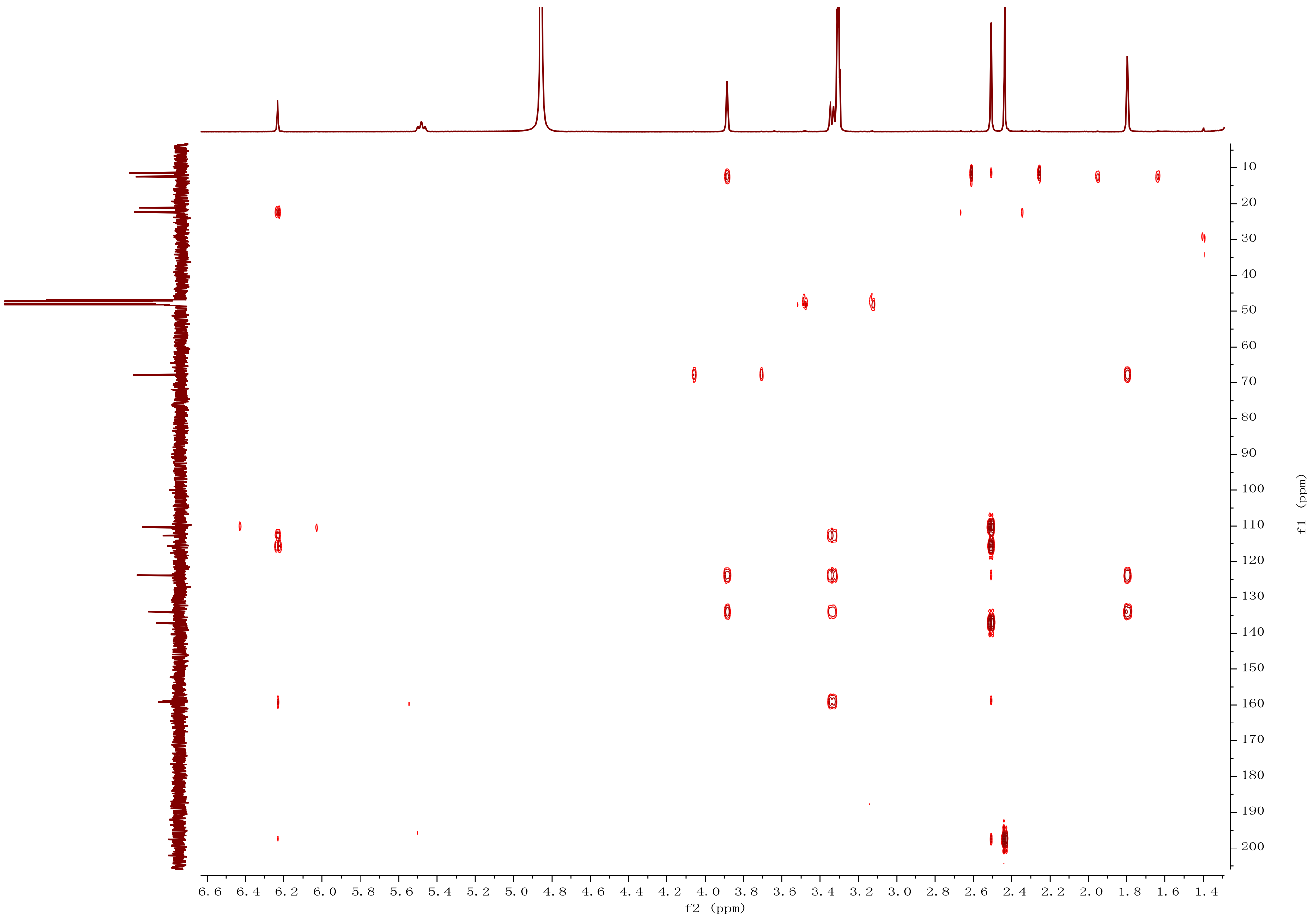

Figure S21. NOESY spectrum of **4** in MeOD.

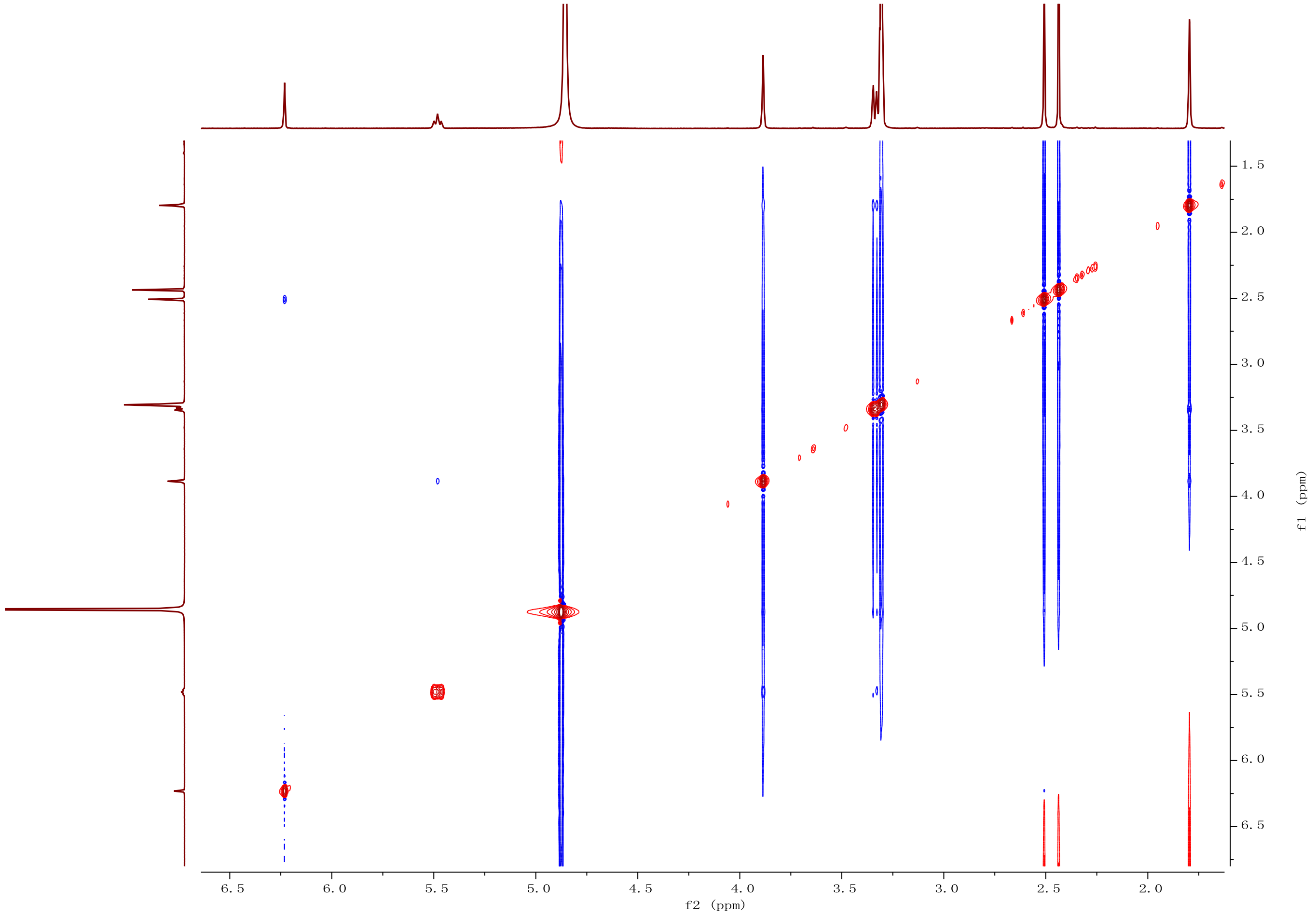

Figure S22. ^{1}H NMR spectrum of **5** in MeOD.

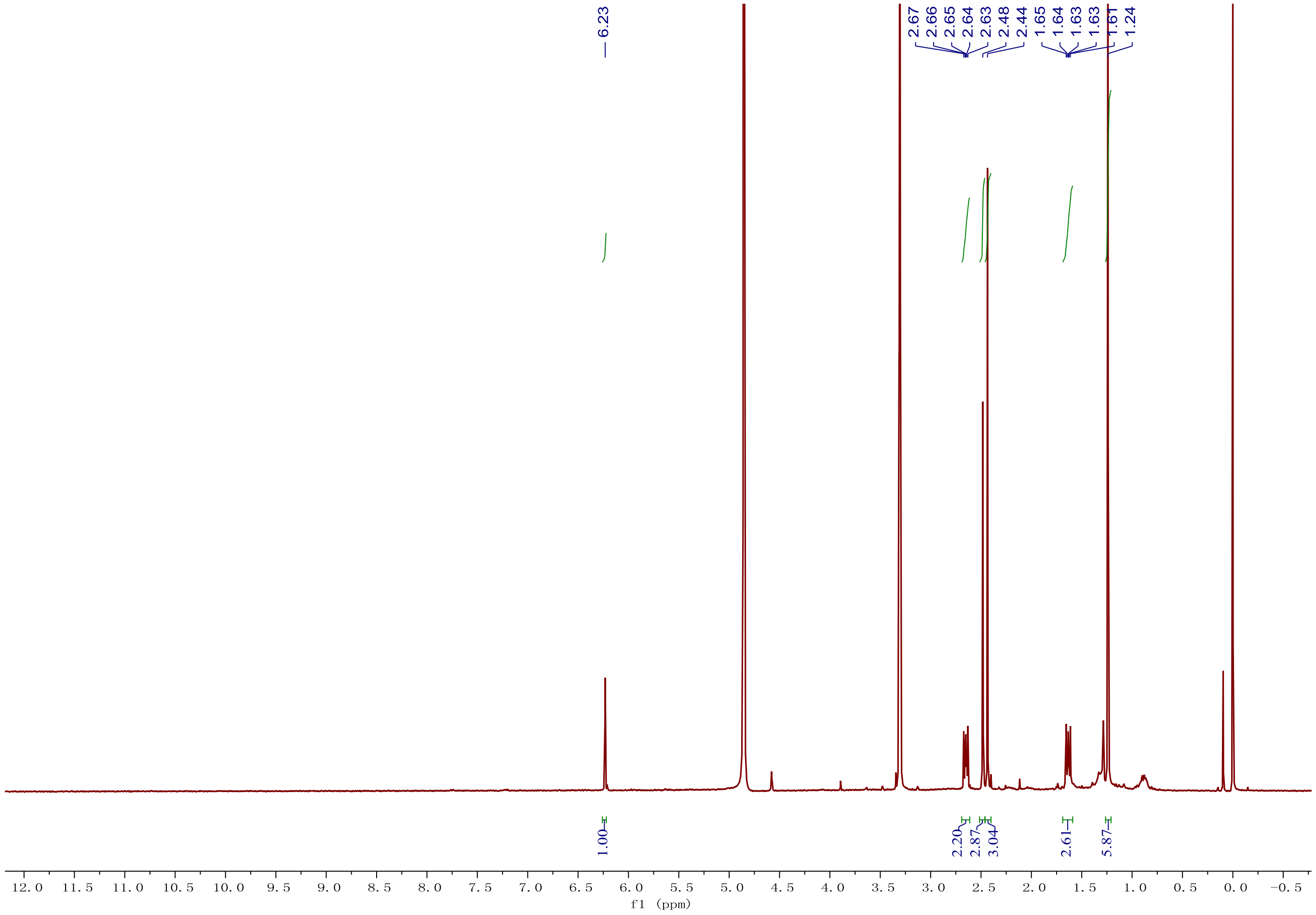

Figure S23. ^{13}C NMR spectrum of **5** in MeOD.

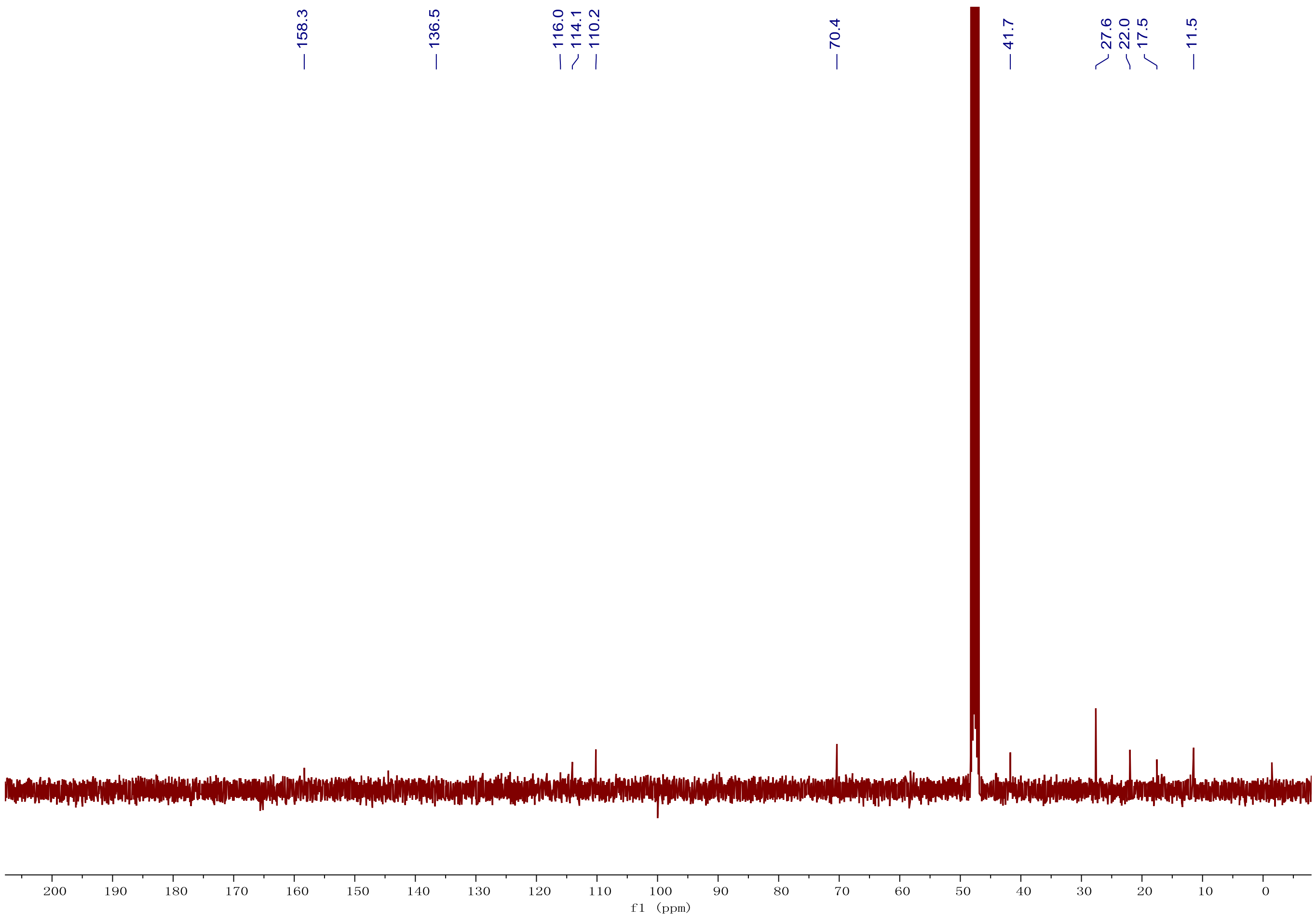

Figure S24. ^{1}H, ^{1}H-COSY spectrum of **5** in MeOD.

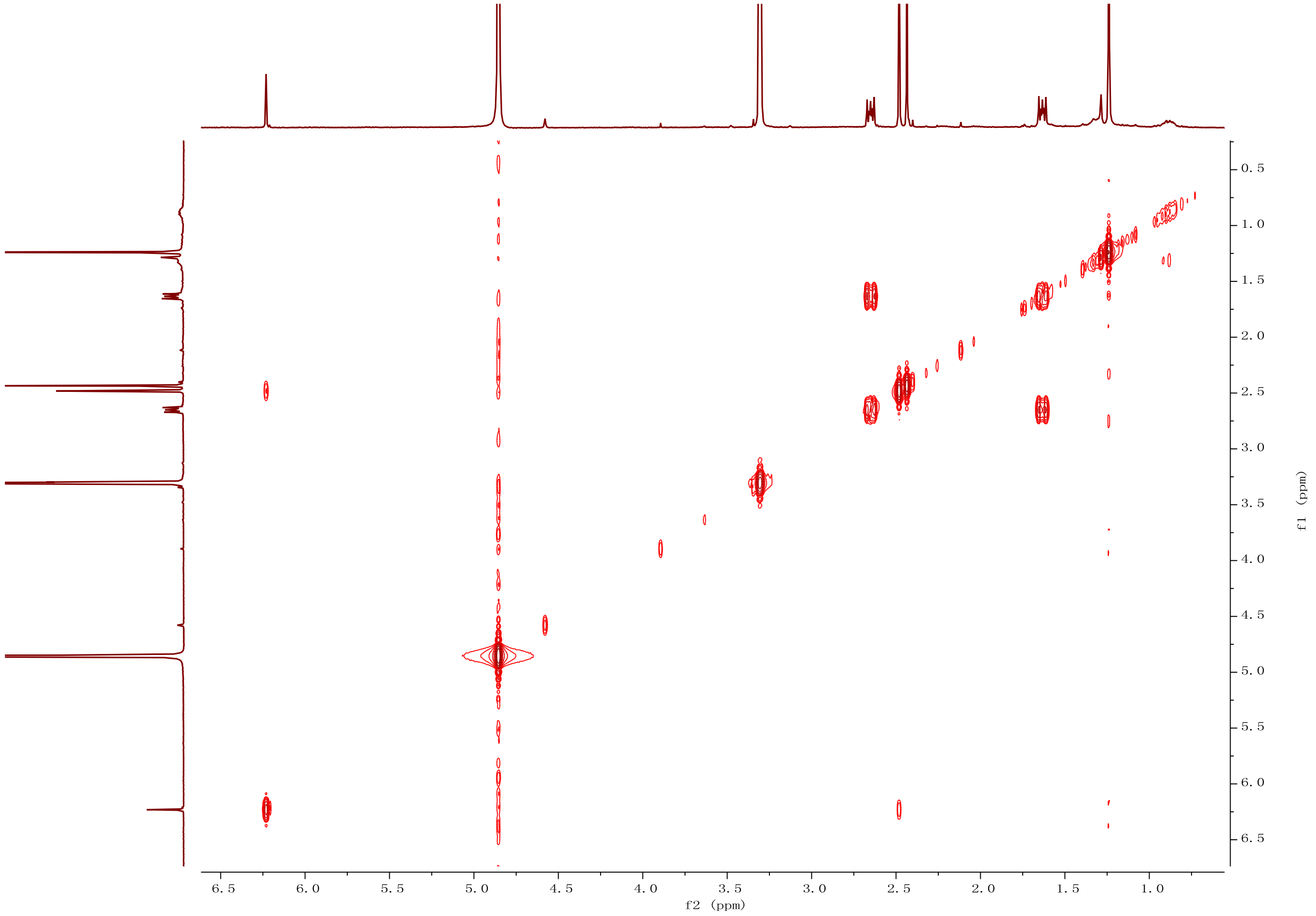

Figure S25. HSQC spectrum of **5** in MeOD.

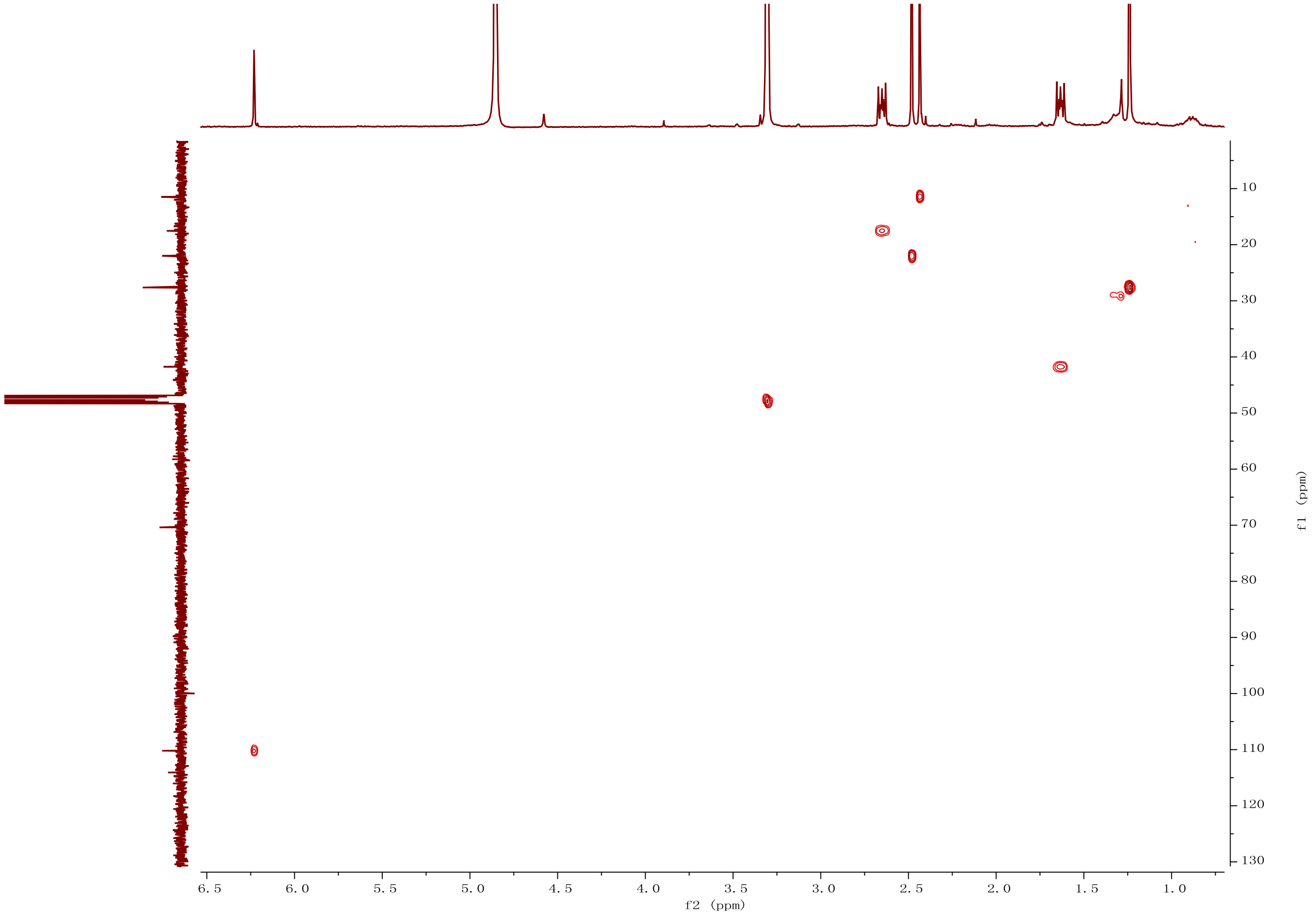

Figure S26. HMBC spectrum of **5** in MeOD.

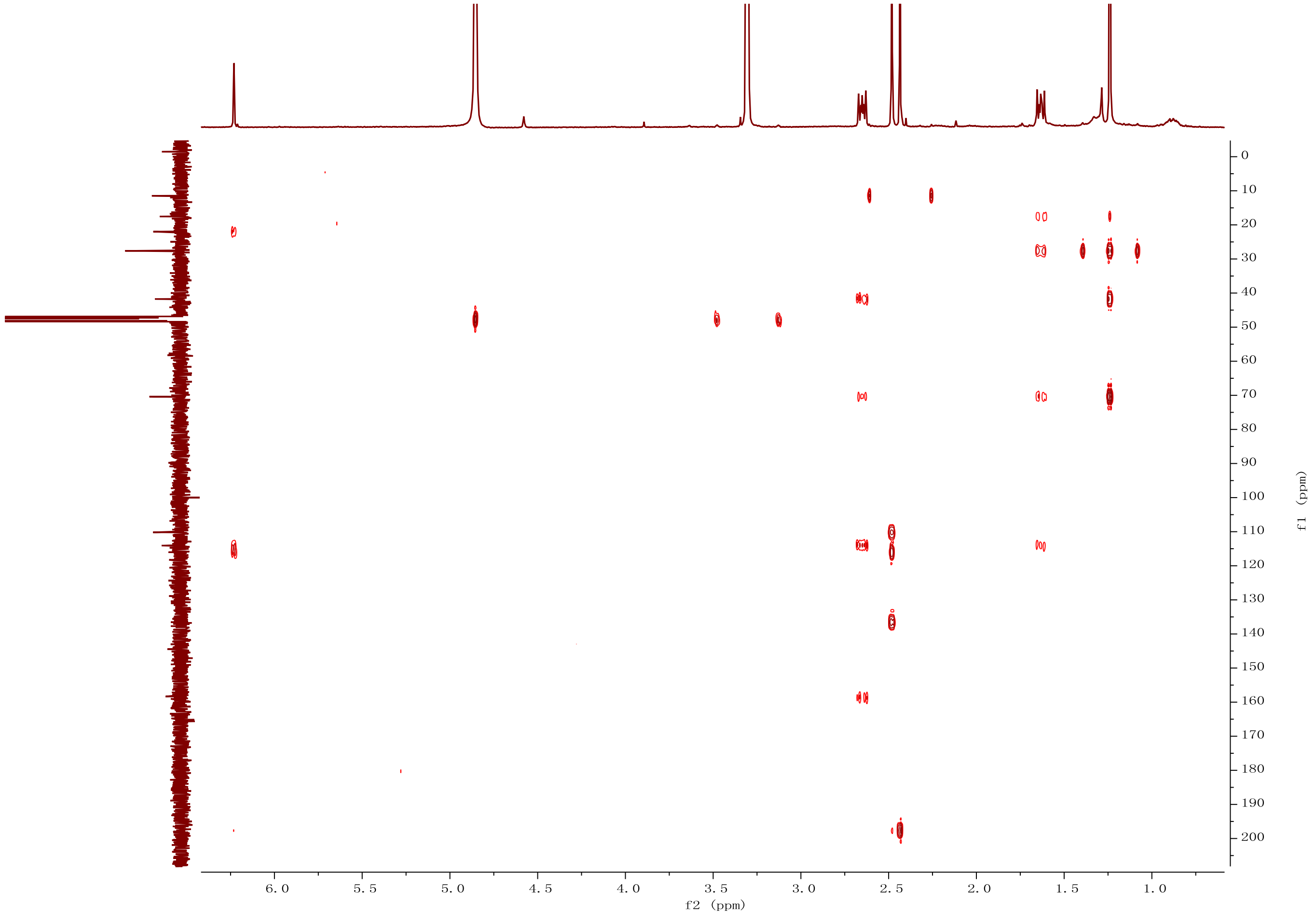

Figure S27. ^{1}H NMR spectrum of **6** in $CDCl_3$.

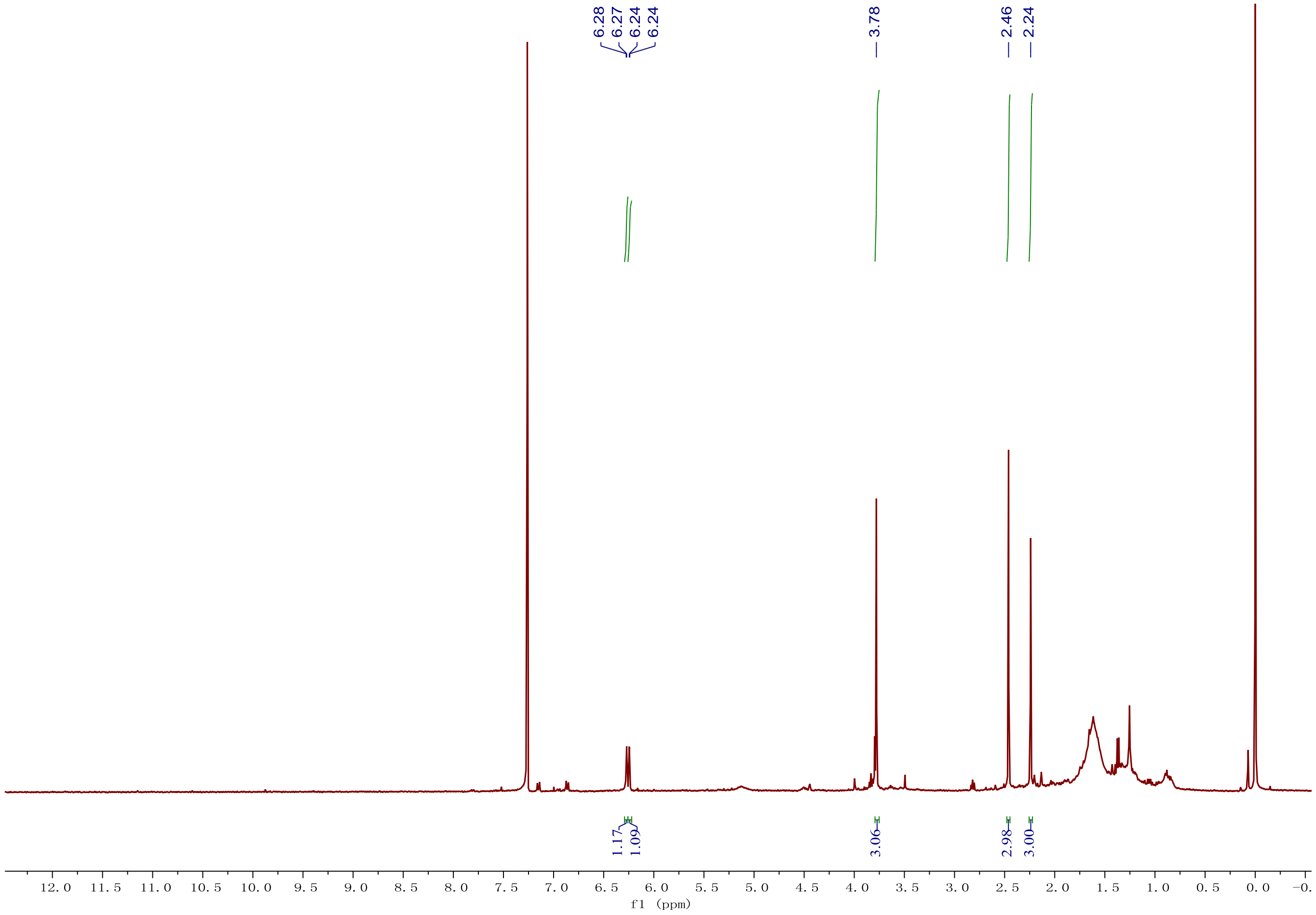

Figure S28. ^{13}C NMR spectrum of **6** in $CDCl_3$.

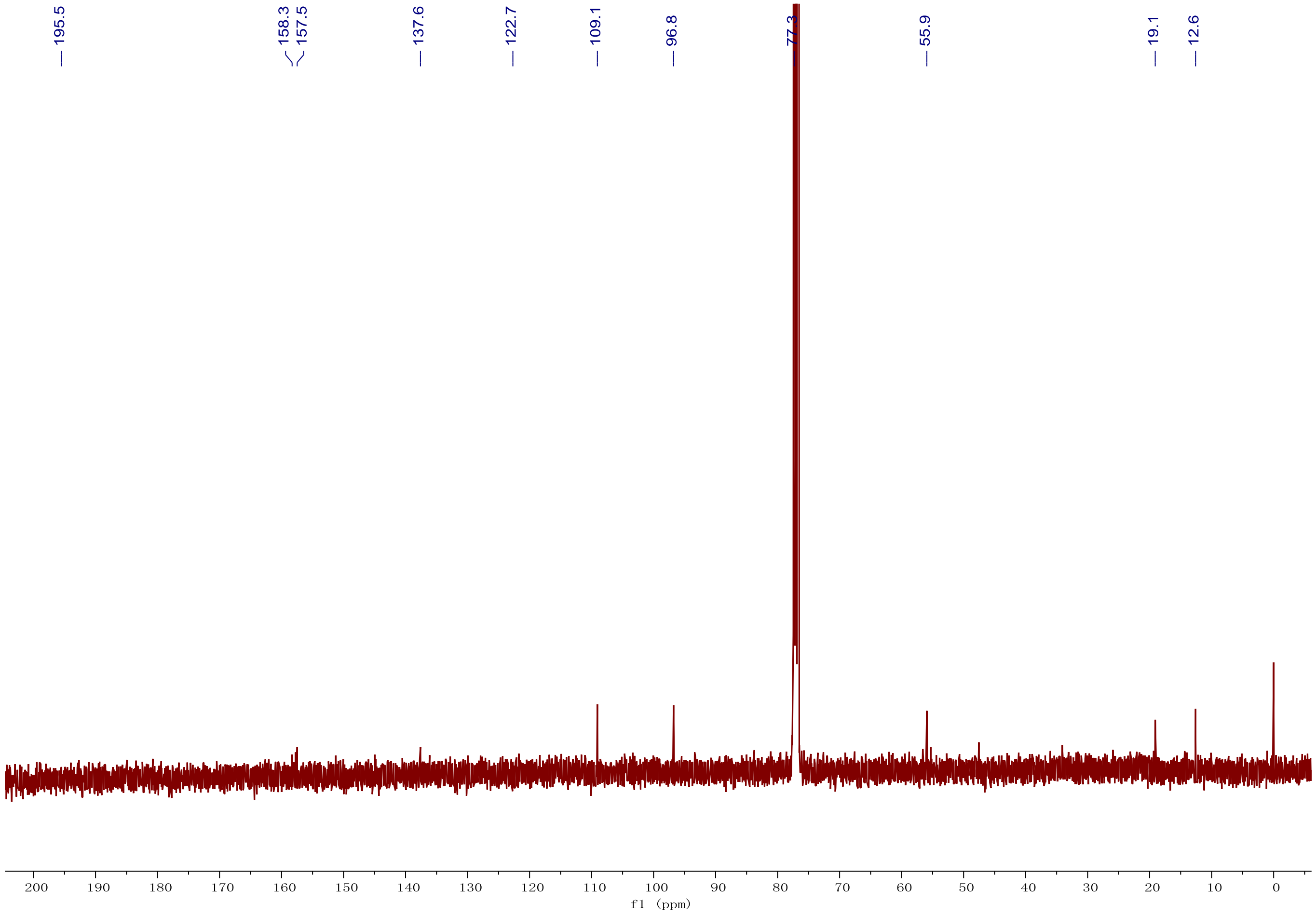

Figure S29. ^{1}H, ^{1}H-COSY spectrum of **6** in $CDCl_3$.

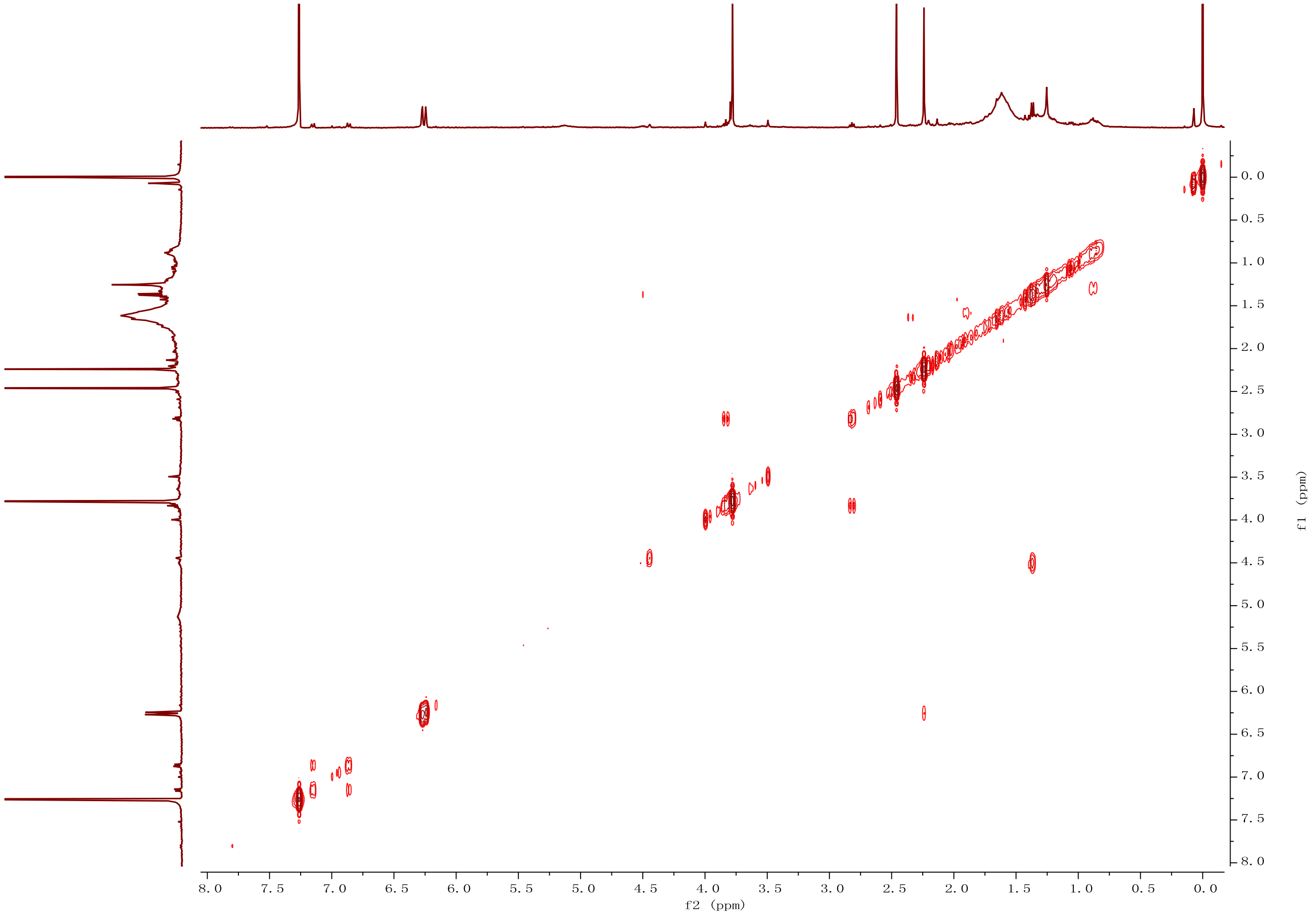

Figure S30. HSQC spectrum of **6** in $CDCl_3$.

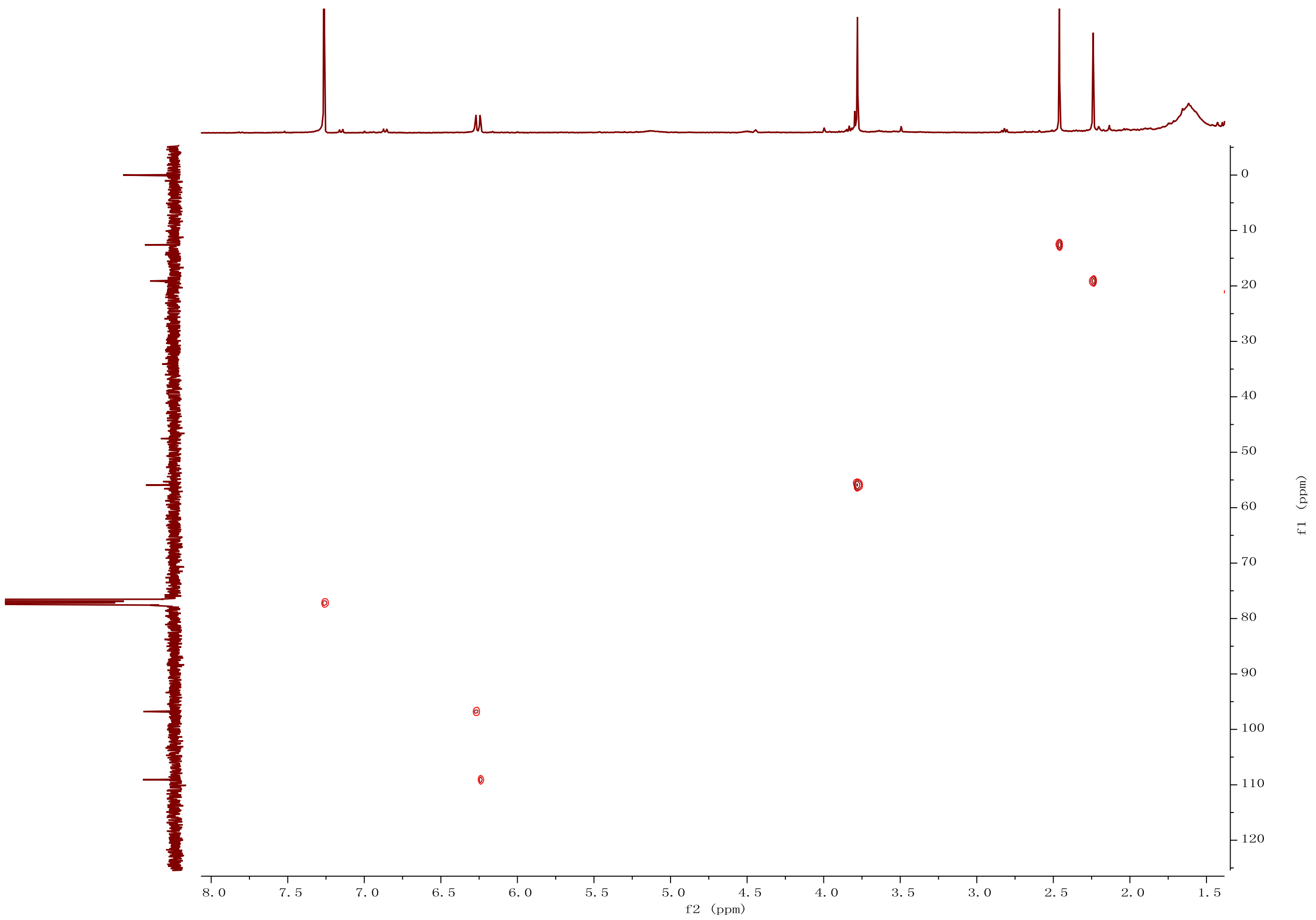

Figure S31. HMBC spectrum of **6** in $CDCl_3$.

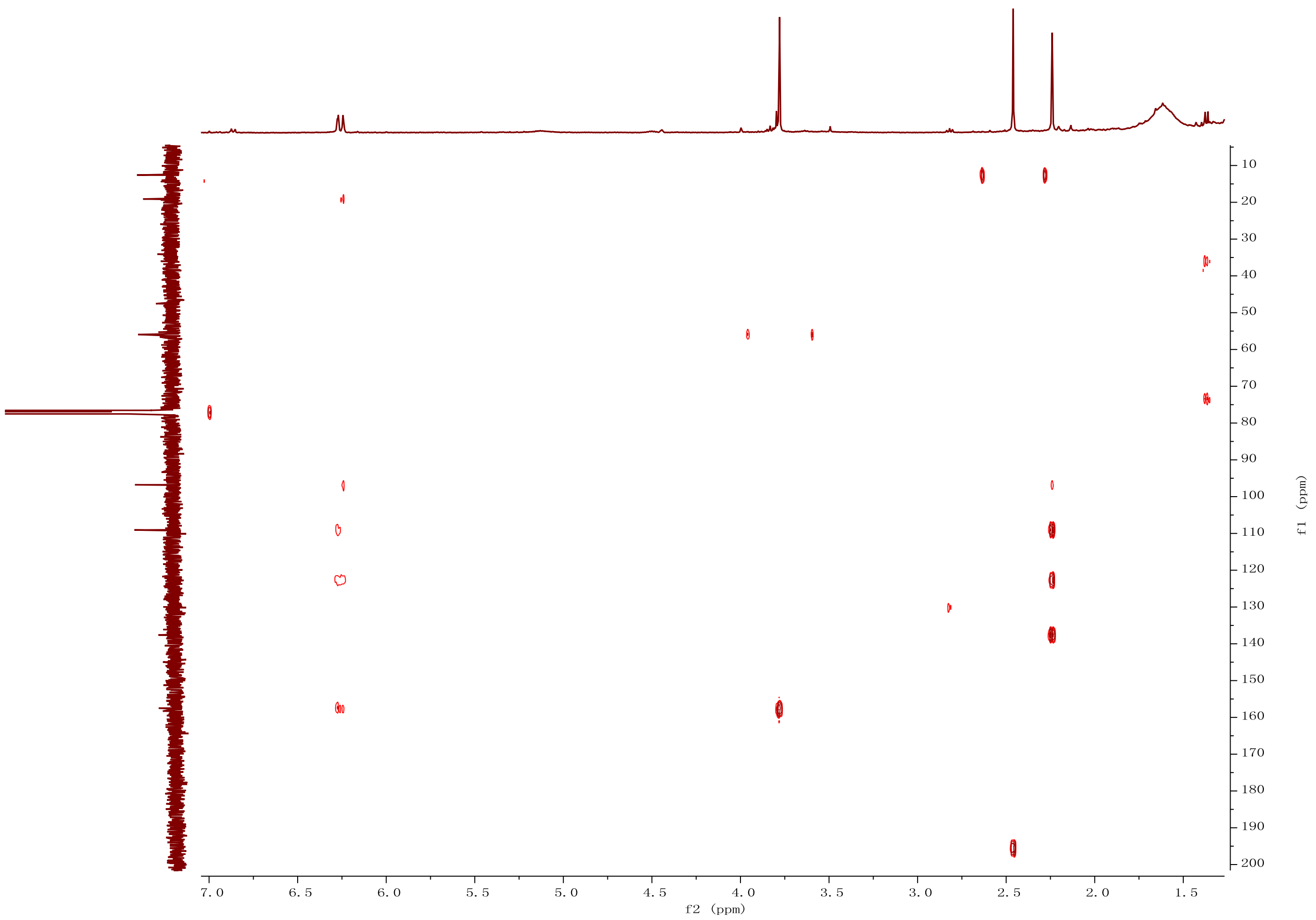

Figure S32. HRESI TOF MS spectrum of **1**.

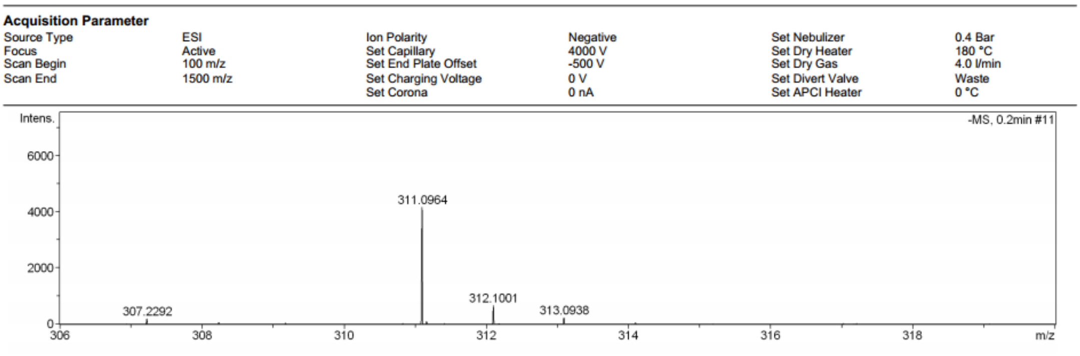

SPECTRUM

- simulation :

m/z	Theo. Mass	Delta (ppm)	RDB equiv.	Composition
311.0964	311.0959	1.6	6.5	C15 H19 O5 S

Limits:

1) Charge: −1
2) Nitrogen-role: Do not use
3) Mass tolerance: 5 ppm
4) Element in use: ^{12}C(0~30), ^{1}H(0~60), ^{16}O(0~10), ^{32}S(0~10)

Figure S33. HRESI TOF MS spectrum of **2**.

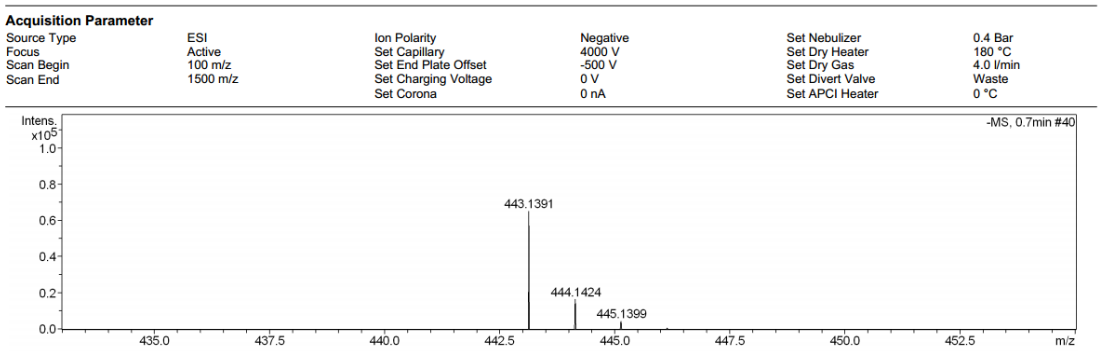

SPECTRUM
- simulation :

m/z	Theo. Mass	Delta (ppm)	RDB equiv.	Composition
443.1391	443.1381	-2.2	7.5	C20 H27 O9 S

Limits:

1) Charge: −1
2) Nitrogen-role: Do not use
3) Mass tolerance: 5 ppm
4) ^{12}C(0~30), ^{1}H(0~60), ^{16}O(0~10), ^{32}S(0~10)

Figure S34. HRESI TOF MS spectrum of **3**.

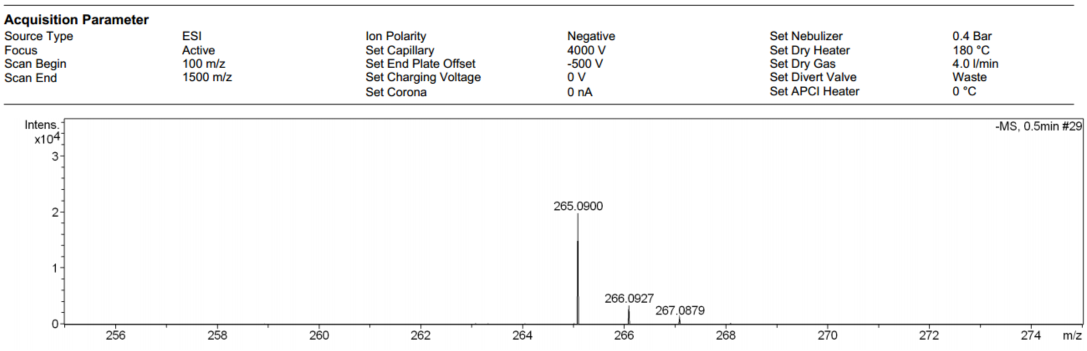

SPECTRUM

- simulation :

m/z	Theo. Mass	Delta (ppm)	RDB equiv.	Composition
265.0900	265.0904	-1.6	6.5	C14 H17 O3 S

Limits:

1) Charge: −1
2) Nitrogen-role: Do not use
3) Mass tolerance: 5 ppm
4) Element in use: ^{12}C(0~30), ^{1}H(0~60), ^{16}O(0~10), ^{32}S(0~10)

Figure S35. HRESI TOF MS spectrum of **4**.

Acquisition Parameter

Source Type	ESI	Ion Polarity	Negative	Set Nebulizer	0.4 Bar
Focus	Active	Set Capillary	4200 V	Set Dry Heater	180 °C
Scan Begin	100 m/z	Set End Plate Offset	-500 V	Set Dry Gas	4.0 l/min
Scan End	1500 m/z	Set Charging Voltage	0 V	Set Divert Valve	Waste
		Set Corona	0 nA	Set APCI Heater	0 °C

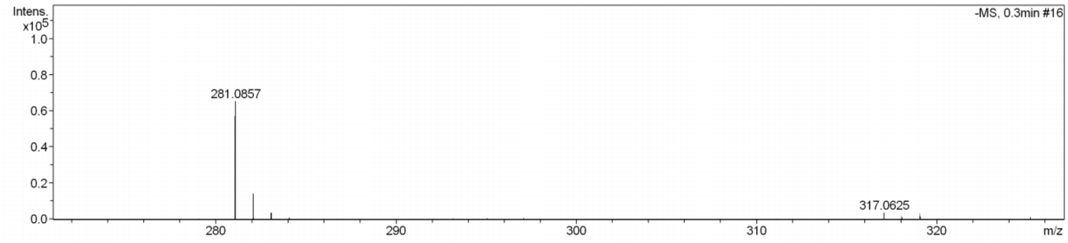

Meas. m/z	#	Ion Formula	Score	m/z	err [ppm]	err [mDa]	mSigma	rdb	e⁻ Conf	N-Rule
281.0857	1	C14H17O4S	100.00	281.0853	1.3	0.4	26.8	6.5	even	ok
317.0625	1	C14H18ClO4S	100.00	317.0620	1.6	0.5	24.5	5.5	even	ok
563.1785	1	C28H35O8S2	100.00	563.1779	1.1	0.6	22.7	11.5	even	ok

SPECTRUM

- simulation :

m/z	Theo. Mass	Delta (ppm)	RDB equiv.	Composition
281.0857	281.0583	1.3	6.5	C14 H17 O4 S

Limits:

1) Charge: −1
2) Nitrogen-role: Do not use
3) Mass tolerance: 5 ppm
4) Element in use: ^{12}C(0~30), ^{1}H(0~60), ^{16}O(0~10), ^{32}S(0~10)

Figure S36. HRESI TOF MS spectrum of **5**.

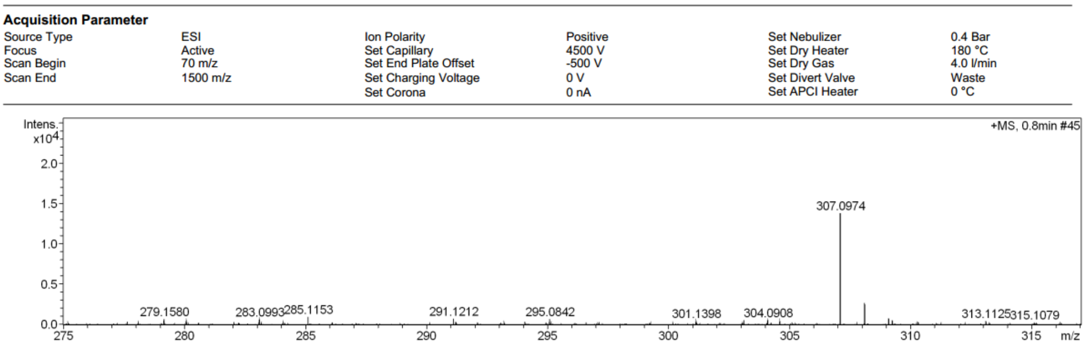

SPECTRUM - simulation :				
m/z	Theo. Mass	Delta (ppm)	RDB equiv.	Composition
307.0974	307.0975	-0.7	4.5	C14 H20 O4 S Na

Limits:

(1) Charge: +1
(2) Nitrogen-Rule: Do not use
(3) Mass tolerance: 5.00 ppm
(4) Elements in use: ^{12}C(0~30), ^{1}H(0~60), ^{16}O(0~10), ^{32}S(0~10)

Figure S37. HRESI TOF MS spectrum of **6**.

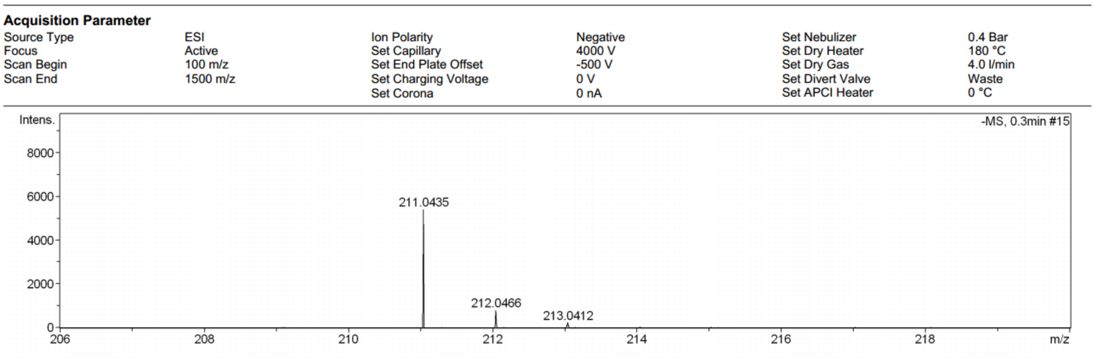

SPECTRUM - simulation :				
m/z	Theo. Mass	Delta (ppm)	RDB equiv.	Composition
211.0435	211.0434	0.1	5.5	C10 H11 O3 S

Limits:

(1) Charge: −1
(2) Nitrogen-Rule: Do not use
(3) Mass tolerance: 5.00 ppm
(4) Elements in use: ^{12}C(0~30), ^{1}H(0~60), ^{16}O(0~10), ^{32}S(0~10)

Figure S38. The observed key NOE correlation between H-10 and H_2-12 in **4**.

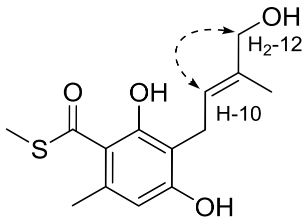

Table S1. Energy analysis for the Conformers of 10*R*-**1**.

compounds	Conformation	G (Hartree)	G (Kcal/mol)	ΔG (Kcal/mol)	Boltzmann Dist (%)
10*R*-**1**	10*R*-**1-1**	-1357.94367667	-852113.5951	0	64.00%
	10*R*-**1-2**	-1357.94275144	-852113.0146	0.58	36.00%

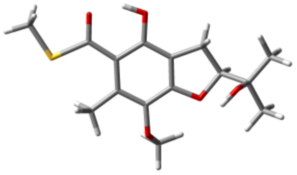

10*R*-**1-1**

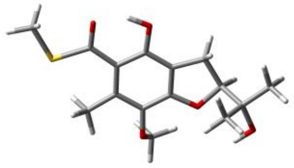

10*R*-**1-2**
